# Microbial removal mechanism of chromium and cadmium by humic acid-loaded nano zero-valent iron prepared by liquid-phase reduction method

**DOI:** 10.3389/fpls.2025.1596063

**Published:** 2025-08-05

**Authors:** Zhiyi Liu, Xuan Zhao, Jianwei Yang, Xi Chen, Yubing Cai, Muhammad Shaaban, Qi-an Peng, Yajun Cai

**Affiliations:** ^1^ School of Resources and Environment, Wuhan Textile University, Wuhan, China; ^2^ Central South Bureau of China Metallurgical Geology Bureau, Wuhan, China; ^3^ College of Agriculture, Henan University of Science and Technology, Luoyang, China; ^4^ Clean Production of Textile Printing and Dyeing Engineering Research Center, Ministry of Education, Wuhan, China

**Keywords:** humic acid, nano zero-valent iron, Cr and Cd pollution, Cr/CdMC, pollution

## Abstract

Heavy metal pollution is a global issue that has drawn significant attention due to its environmental and health risks. This thesis focuses on the research of highly toxic chromium and cadmium in the environment. It explores the removal mechanism of Cr and Cd contamination using humic acid-loaded nano-zero-valent iron (NZVI@HA) prepared through a liquid-phase reduction method. Additionally, it investigates the interaction mechanism of removing Cr and Cd contamination by synergizing with the Chromium and Cadmium Symbiotic Bacterial Colony (NZVI@HA+Cr/CdMC). The findings indicate that NZVI@HA exhibited optimal removal efficiency for Cr(VI) at pH=2 (85.7%) and Cd(II) at pH=8 (94.8%). The initial concentration of Cr and Cd pollution showed an inverse relationship with the removal rates of Cd(II) and Cr(VI). Moreover, the reaction temperatures were positively correlated with the removal rates of Cd(II) and Cr(VI). Cu2+ significantly enhanced Cr(VI) removal in the water column (p<0.01), whereas Zn2+ notably inhibited Cd(II) removal (p<0.05). In the NZVI@HA+Cr/CdMC system, extracellular polymers (EPS), tyrosine, and tryptophan, through van der Waals forces, facilitated the removal of Cd(II) and Cr(VI) complexation. This reduced the stress of Cr(VI) and Cd(II) on Cr/CdMC, thereby enhancing the removal of Cr(VI) and Cd(II).

## Introduction

Chromium, a redox-active 3d transition metal, primarily exists in two oxidation states: trivalent and hexavalent. Cadmium is predominantly found in a divalent state. The environmental pollution of Cd(II) and Cr(VI) poses significant risks to human health and ecosystems (Jaiswal et al., 2018). Cr(IV) specifically can lead to various health issues, including lung cancer, liver damage, kidney failure, weakened immune system, respiratory problems, stomach ulcers, and skin rashes (Jiang et al., 2021). Cd(II) exposure has been linked to kidney damage, bone fragility, lung damage, and cancer (Haider et al., 2023). According to the Manual of Total Environmental Exposure and Contribution Ratio of Metals for Residents in Typical Areas published by the Chinese Academy of Environmental Sciences in 2019, the key sources of environmental contact to Cr(VI) and Cd(II) for surveyed residents are primarily dietary, accounting for 61.23% to 99.77%. Cd and Cr can travel through the soil-water-rice-human body system, leading to bioaccumulation in the human body and resulting in severe diseases such as prostate, liver, and kidney cancers. Both heavy metals accumulate in the environment, causing harm to ecosystems including aquatic and terrestrial organisms (Parida and Patel, 2023).

The co-contamination of Cr(VI) and Cd(II) is prevalent in industrial activities such as electroplating, tanning, mining, smelting, and agricultural practices. For instance, in electroplating and tannery wastewater, Cr(VI) serves as an oxidizing agent for coating deposition, while Cd(II) originates from metal processing or pigment additives. In mining and smelting operations, the geochemical association of chromium-cadmium results in their simultaneous release. In agriculture, prolonged use of chromium-containing pesticides and cadmium-contaminated fertilizers leads to the accumulation of both heavy metals in soil systems (Tang et al., 2014). These contaminants exhibit synergistic toxicity due to their distinct physicochemical properties: Cr(VI), with its strong oxidizability and high mobility, induces oxidative stress that disrupts cellular membrane integrity, thereby enhancing the transmembrane permeability of Cd(II). Conversely, Cd(II), characterized by bioaccumulation potential and persistent toxicity, suppresses antioxidant enzyme activities (e.g., superoxide dismutase), exacerbating the genotoxic effects of Cr(VI), such as DNA damage (de Melo et al., 2016). The coexistence of these pollutants in environmental matrices may trigger speciation transformation (e.g., Cr(VI)/Cr(III) redox coupling), competitive adsorption on remediation substrates, and inhibition of bioremediation efficacy. Consequently, conventional single-pollutant remediation strategies fail to address these complexities, necessitating the development of synchronized removal technologies and synergistic regulation approaches to achieve efficient environmental restoration (Li et al., 2017).

In current years, the research has focused on the elimination of Cd(II) and Cr(VI) from water bodies using nano-zero-valent iron(NZVI)-based materials (Huang et al., 2024). **Table 1** provides a summary of current studies on NZVI and its composites for removing Cd(II) and Cr(VI) from aqueous environments. Additionally, there has been an uptick in studies on the co-removal of pollutants in binary systems. For instance, Zhao et al. (2022) investigated the mechanism and consequence of co-removing Cd(II) and Cr(VI) using biochar loaded with sulfide-modified NZVI in dual systems. Their findings indicated that the presence of Cd could enhance the elimination of Cr, though the presence of Cr could hinder the amputation of Cd. Furthermore, Liu et al. (2021) studied the reduction and adsorption of U(VI) and Cr(VI) using NZVI loaded onto poly(dopamine)-modified iron nanoparticles. Their results demonstrated that U(VI) and Cr(VI) initially adhered to the composite surface through electrostatic interactions or surface complexation reactions. Subsequently, approximately 55.7% of Cr(VI) and 47.1% of U(VI) were reduced to Cr(III) and U(IV).

**Table 1:** Latest research on the elimination of Cd (II) and Cr (VI) from water environments by NZVI and its composite materials.

Materials	Reaction Condition	Removal performance	References
Novel amino-modified bamboo-based biochar loaded with nano zero-valent iron	pH=5, Cr(IV)=30 mg L^-1^, Dosage applied=0.5 g L^-1^	95.3%	(Zhou et al., 2023)
Zero-valent iron modified tea-dregs derived porous biochar	pH =6, Cr(IV)&Cd(II)=10 mg L^-1^, Dosage= 10mg	186.2mg/g 19.1 mg/g	(Rajput et al., 2023)
S-nZVI loaded with chitosan and biochar	pH =5, Cd(II)=50mg L^-1^, Dosage applied=0.2 g L^-1^	249.92 mg/g	(Li et al., 2024)
Sulfide modification of calcium alginate loading nZVI	pH =6, Cr(IV)&Cd(II)=20 mg L^-1^, Dosage applied= 0.2 g L^-1^	4.57 mg/g	(Liang et al., 2022)
Carbon-based nano zero-valent iron complexes of drug residue	pH =4, Cr(IV)=40 mg L^-1^, Dosage applied=1.5 g L^-1^	97%	(Diao et al., 2023)
Zero-valent iron nanocomposites from kaolinite	pH =4, Cr(IV)=20mg L^-1^, Dosage applied= 2 g L^-1^	99.6%	(Dai et al., 2023)
Chitin microsphere-loaded sulfurized nano zero-valent iron	pH =4, Cr(IV)=20mg L^-1^, Dosage applied=1 g L^-1^	924.5 mg/g	(Chen et al., 2023)
Biochar-loaded sulfide-modified NZVI	pH =5, Cr(IV)&Cd(II)=50 mg L^-1^, Dosage applied= 1 g L^-1^	58.87 mg/g 32.55 mg/g	(Zhao et al., 2022)
Polydopamine-modified SBA-15-loaded nano-zero-valent iron	pH =2, Cr(IV)=3 mg L^-1^, Dosage applied=10 mg	83%	(Liu et al., 2021)

Humic acid (HA) is a macromolecule consisting of humic substances (HS) with non-homogeneous and porous complex reticulated or lamellar structures (Francioso et al., 2019). These organic compounds are found in natural waters, terrestrial soils, and sediments (de Melo et al., 2016). HA, being a naturally occurring organic carbon skeleton, is abundant in functional groups like carboxyl, phenolic hydroxyl, and methyl groups. These properties enable it to enhance soil fertility, boost crop yields, and aid in pollutant removal (Zhu et al., 2023). The incorporation of NZVI onto HA enhances the dispersion and stability of NZVI (Zeng et al., 2024), effectively preventing agglomeration and passivation of NZVI (Yao et al., 2019; Wu et al., 2025). Additionally, the occurrence of HA promotes the electron transfer process of NZVI, enhancing its reduction capabilities when combined as NZVI@HA (Liang et al., 2024). This synergy makes NZVI@HA a highly efficient and environmentally friendly material with diverse applications, showing significant promise in the realm of environmental remediation (Fu et al., 2017).

Furthermore, an increasing number of researchers and scholars have reported on the elimination of Cr(VI) by Cr-reducing bacteria (Zeng et al., 2023). For instance, Sahoo et al. (2023) isolated the native rhizobacterium CR02 from the water of a chromite quarry in the Sukinda Valley, India, for Cr(VI) reduction. The CR02 strain displayed high tolerance to Cr levels up to 520 mg/L, with its chromate reductase playing a key role in the reduction process. Li et al. (2022) utilized NO_3_^–^mediated NZVI with mixed anaerobic microbes for Cd(II) elimination studies. They found that nitrate-mediated bioFe0 columns had improved Cd(II) amputation efficacy due to the introduction of microorganisms, leading to the generation of more Fe(II), Fe(III), and secondary minerals that facilitated Cd(II) adsorption and immobilization. Moreover, Mohamed et al. (2020) explored the Cr(VI) removal by Shewanella oneidensis MR-1 in the presence of humic acid (HA) and acicular ferrite, demonstrating that Fe mineral-HA complexes improved Cr(VI) microbial removal. Long et al. (2019) observed that most of the Cr was located in the extracellular polymer (EPS) and cell wall, with EPS exhibiting higher Cr(VI) reduction compared to the cell wall, leading to the development of Cr(III) in the supernatant post-reduction. Additionally, Le et al. (2023) discovered that an innovative Cr(VI)-reducing anaerobic bacterium, *Paraclostridium bifermentans* G3, could form a CdS-Paracostridium complex on the cell surface, enhancing the Cr(VI) reduction process significantly.

In summary, microorganisms can independently reduce Cd(II) and Cr(VI) from the environment and can also collaborate with materials to enhance the removal of Cr(VI) and provide additional reaction sites for the Cd(II) and Cr(VI) confiscation, thereby aiding in the elimination of Cd(II) and Cr(VI) contamination. This study primarily focused on investigating the Cd and Cr elimination using humic acid-loaded NZVI (i.e. NZVI@HA) under various situations (Xia et al., 2021).

Conventional nanoscale zero-valent iron (NZVI) has been widely employed for heavy metal remediation owing to its strong reductive capacity and high reactivity. However, inherent limitations such as particle agglomeration, surface passivation, and poor environmental adaptability severely restrict its practical efficiency. Thus, the development of novel composite materials capable of stabilizing NZVI dispersion while synergistically enhancing its functionality is urgently required (Duan et al., 2023; Wu et al., 2025).

Humic acid (HA), a natural organic macromolecule enriched with carboxyl (-COOH), hydroxyl (-OH), and carbonyl (-CHO) functional groups, not only directly participates in heavy metal removal through complexation, adsorption, and reduction mechanisms but also serves as an effective carrier to regulate the dispersibility and stability of nanomaterials. This study proposes HA-supported NZVI (NZVI@HA) to address the agglomeration and passivation of NZVI from a chemical perspective while leveraging HA’s multifunctional properties to synergistically enhance Cr(VI) and Cd(II) removal.

Notably, HA incorporation not only directly improves material performance but also amplifies redox and complexation processes through extracellular polymeric substances (EPS). Functional groups in tyrosine and tryptophan (e.g., -OH and π-π interactions) enable tyrosine to act as a reductant for Cr(VI) detoxification and tryptophan to form stable complexes with Cr(VI)/Cd(II) via van der Waals forces (Hou et al., 2019). Furthermore, NZVI@HA stimulates metabolic activity and upregulates reduction-related gene expression in chromium-cadmium co-contaminated microbial consortia (Cr/CdMC), enhancing their tolerance to heavy metal stress and indirectly promoting contaminant removal. This multi-mechanistic synergy establishes a novel chemico-biological remediation strategy for composite pollution, underscoring the scientific and practical significance of HA-supported NZVI in environmental functional material design.

Among existing technologies, NZVI stands out for its strong reductive capacity and high reactivity, yet its agglomeration and stability issues necessitate further optimization. HA, a naturally abundant and biodegradable material, offers exceptional adsorption capabilities without introducing secondary pollutants, thereby preserving ecological balance. Microbial consortia, capable of simultaneously or sequentially removing multiple heavy metals under natural conditions, represent a sustainable remediation approach (Gola et al., 2020). Despite these advantages, studies integrating NZVI@HA with microbial systems to enhance Cr(VI) and Cd(II) removal remain scarce. This knowledge gap highlights the critical need to explore the chemico-biological synergy between HA-stabilized NZVI and microbial communities. This study bridges critical research gaps by elucidating the multi-mechanistic synergy of NZVI@HA in chemico-biological systems, offering a sustainable strategy for composite pollution remediation. The integration of HA’s eco-friendly properties, NZVI’s reactivity, and microbial metabolic resilience not only addresses NZVI’s limitations but also advances the design of environmentally functional materials for real-world applications.

The study examined the Cd(II) and Cr(VI) removal from contaminated environments using NZVI@HA, Cr/CdMC, and NZVI@HA+Cr/CdMC systems. Various analytical techniques such as microbial scanning electron microscopy (SEM), XPS, 3D-EEM, high-throughput sequencing, and fluorescence quantitative PCR were utilized to examine the interaction mechanism of NZVI@HA and Cr/CdMC in removing Cd(II) and Cr(VI).

## Materials and methods

### Purification of humic acid

Crude humus weighing 20.0 ± 0.5 g was placed in a 1000 mL beaker, and 400 mL of 0.1 mol/L NaOH solution was added. The beaker was then positioned on a magnetic stirrer at 5000 rpm for 24 hours. Subsequently, the mixture was transferred into 100 mL centrifuge tubes and subjected to high-speed freezing centrifugation at 6500 rpm for 5 minutes. The supernatant was collected in a 500 mL beaker, and the pH was adjusted to approximately 1 using 6 mol/L hydrochloric acid, allowing it to sit for 24 hours. Afterward, the supernatant was poured out, and the remaining precipitates were washed thrice with 0.1 mol/L HCl. The purified humic acid (HA) was then ground using an onyx mortar, sieved through a 100-mesh nylon sieve, and stored under vacuum for future use.

### Preparation of humic acid-loaded NZVI

The HA-loaded NZVI (i.e. NZVI@HA) was synthesized through liquid-phase reduction. Purified HA and ferrous sulfate heptahydrate (FeSO_4_-7H_2_O) were combined in a wide-mouth flask in varying ratios [HA: Fe(II)] of 1:2, 1:1, 2:1, 3:1, and 4:1. Subsequently, 100 mL of deoxidized purified water was added, and the flask was placed on a shaker at 150 rpm for 12 hours to ensure uniform mixing of HA and FeSO_4_-7H_2_O. The mixture was then shifted to a 3-necked flask under nitrogen gas flow, and a sodium borohydride solution was added drop by drop using a dispenser funnel. The reaction continued under a nitrogen atmosphere for 10 minutes to ensure complete reduction of Fe(III) and Fe(II) to Fe0, resulting in a solution with a black precipitate.

The three-necked flask was sealed under nitrogen protection, and the solution was separated by magnet adsorption. The supernatant was decanted, and the resulting precipitates were washed thrice with cold water and thrice with anhydrous ethanol. This process yielded NZVI@HA in different ratios.

### Study of Cd and Cr amputation by NZVI@HA

To examine the impact of the HA: Fe(II) ratio on Cd(II) and Cr(VI) elimination, various HA: Fe(II) mass ratios (1:1, 1:2, 2:1, 3:1, and 4:1) were examined. The amount of the HA: Fe(II) mixture we used was 2 g/L. The study assessed the removal of Cd(II) and Cr(VI) from Cd and Cr contamination. Additionally, the research explored the influences of pH, initial concentration, temperature, and competitive ions on the Cd(II) and Cr(VI) elimination of from Cd and Cr pollution using NZVI@HA. The detailed preparation process and characterization methods were presented in Text S1, respectively.

### Enrichment of Cr/Cd symbiotic flora

Twenty grams of Cd and Cr polluted soils were weighed and added to a sterilized conical flask containing 150mL of ultrapure water. The mixture was shaken well and placed in a constant temperature incubator at 25°C for 48 hours. Subsequently, 10mL slurry (precise volume) was inoculated into a conical flask (sterilized) containing 150mL of LB medium, shaken well, and shifted to a constant temperature (25°C) incubator for 7 days until reaching an optical density of 600 (OD600 greater than 1). Ten milliliters of the microbial bacterial liquid were then inoculated into a conical flask containing 150mL of Cr and Cd medium (initial Cr and Cd concentration of 25 mg/L). The mixture was shaken well and placed in a constant temperature (25°C) incubator for 7 days (OD600>1). Subsequently, each concentration was increased up to 200 mg/L (each with an increment of 50 mg/L) being incubated for 7 days till reaching Cr(VI) and Cd(II) mass concentration of 200mg/L. Three similar groups were established for individual concentration. The enriched Cr/Cd symbiotic flora was denoted as Cr/CdMC.

### Elimination of Cd(II) and Cr(VI) by NZVI@HA synergized Cr/CdMC

A conical flask (250 mL) containing 80mL of LB liquid medium and a 1 g/L Cr and Cd mixed solution underwent sterilization in an autoclave at 121°C for 20 minutes. After cooling, 20 mL of the sterilized 1 g/L Cr&Cd mixed solution was added to the sterilized LB liquid medium to create a 200 mg/L medium containing Cr and Cd. The pH of the Cr and Cd-containing medium was maintained at a natural pH range of 6.8 to 7.1. All experiments were conducted under aseptic conditions to prevent contamination by stray bacteria and minimize errors.

Four treatments were established: Cr&Cd-added medium (CK), humic acid (HA)-loaded nano zero-valent iron (NZVI@HA), Cr/Cd symbiotic colony (Cr/CdMC), and a system combining HA-loaded NZVI with Cr/Cd symbiotic colony (NZVI@HA+Cr/CdMC). Each treatment consisted of three parallel groups. The starting concentrations of Cd and Cr were both set at 200mg/L. The selected dosage of NZVI@HA was 1 g/L, and the inoculum of the Cr/Cd symbiotic bacterial colony (Cr/CdMC) was 2% (v/v). The samples were placed at 25°C for 7 days, with samples taken and analyzed daily. Cr(VI) determination was performed using the dibenzoyl dihydrazide spectrophotometric method, while inductively coupled plasma emission spectrometry was utilized for the determination of Cd(II) and the measurement of dissolved Fe content.

### Characterization

The microbial characterization of the Cr/CdMC and NZVI@HA+Cr/CdMC systems after 7 days of incubation involved SEM, EDS, and excitation emission matrix spectroscopy (3D-EEM) to analyze changes in elemental composition, valence, and extracellular polymers (EPS) of the chromium and cadmium symbiotic colonies.

For EPS extraction, 30mL of bacterial solution was shifted to a 100mL centrifuge tube and subjected to high-speed freezing centrifugation at 4°C, 6000 rpm for 5 min. The supernatant was discarded, and 0.85% NaCl solution was added to the original volume. This process was repeated in the same way three times, and the cleaned precipitate was sonicated and heated for 30 minutes in an ultrasonic device. Subsequently, the mixture was centrifuged at 4°C, and the supernatant was passed through a 0.45µm membrane to obtain EPS.

The determination of EPS was conducted using a fluorescence spectrophotometer with the following parameters: excitation wavelength (EX) and emission wavelength (EM) ranging from 200 to 600 nm, an amplitude of 5 nm, and excitation and emission slit widths of 3.6 nm.

### Microbiological analysis

High-throughput sequencing: The bacterial liquid post-reaction was shifted to a sterilized 15mL centrifuge tube, frozen, and stored at -4°C before being dispatched to Personalbio for analysis. The analysis utilized the partial sequence of the 16S_V3V4a gene with universal primers (F: ACTCCTACGGGGAGGCAGCA, R: GGACTACHVGGGTWTCTAAT). The initial denaturation amplification conditions were set at 98°C for 2 min, cycling amplification conditions at 98°C for 15 sec, 55°C for 30 sec, and 72°C for 30 sec, with final amplification conditions at 72°C for 5 min.

Fluorescence quantitative PCR: After reaction, the bacterial liquid was stored in a sterilized 15 mL centrifuge tube at -4°C and then forwarded to Personalbio for analysis. The primer details used in the analysis are provided in **Table 2**.

**Table 2:** Primer information used in this experiment.

Gene name	Forward primers (5’- 3’)	Reverse primers (5’- 3’)
CzcA	TCGACGGBGCCGTGGTSMTBGTCGAGAA	GTVAWSGCCAKCGGVBGGAACA
NitR	GACACCCGCCCGCATCTCAT	TGTCCCAGTCGCCTTCCACC
ChrA	GAATGCGCCCATGAAACC	TAGCACCTGTCGTTCTGTT

## Results and discussion

### Characterization of NZVI@HA

The removal effects of NZVI@HA on Cd(II) and Cr(VI), prepared with varying ratios of HA and Fe(II), are illustrated in **Supplementary Figure S1**. The adsorption potential of Fe(II) on Cr(VI) initially exhibited a slow increasing trend, peaking at 73 mg/g when HA: Fe(II) was 3:1. Subsequently, both the removal effect and adsorption capacity declined. In contrast, the adsorption capacity of Fe(II) on Cd(II) displayed a linear upward trajectory, reaching a highest of 215 mg g^-1^, with a unit mass adsorption capacity of 180 mg g^-1^ for Fe(II) at a HA: Fe(II) ratio of 3:1. Considering economic factors and the overall removal efficiency of NZVI@HA on Cd(II) and Cr(VI), a synthesis ratio of HA: Fe(II) of 3:1 was chosen for preparing NZVI@HA.

Scanning electron microscopy (SEM) analysis depicted the surface morphology of HA, NZVI, and NZVI@HA, as depicted in **Supplementary Figure S2**. The SEM results validated the successful loading of NZVI onto the HA surface, with an overall particle size below 70 nm and no significant agglomeration.

The surface functional groups of humic acid, NZVI@HA, and NZVI were examined using Fourier transform infrared spectroscopy (FTIR), as depicted in **Supplementary Figures S3A, B**. Various characteristic peaks in the figures represent distinct functional groups. The primary functional groups of HA include carboxyl group (COOH), phenol hydroxyl/hydroxyl group (OH), aldehyde group (CHO), and carbon-carbon triple bond (C≡C). The characteristic peak at 1704 cm^-1^ corresponds to the vibration and stretching of the carboxyl group (COOH), while peaks at 3373 cm^-1^ and 1379 cm^-1^ represent phenol hydroxyl and hydroxyl (OH) stretching vibrations, respectively. The peak at 1613 cm^-1^ indicates aldehyde (CHO) stretching vibration, and peaks at 2340 cm^-1^ and 2361 cm^-1^ signify C≡C stretching vibration.

In comparison to HA, NZVI and NZVI@HA exhibited distinctive iron characteristic peaks. The peak at 539 cm^-1^ corresponds to Fe-O stretching vibration, 3687 cm^-1^ to Fe-OH stretching vibration, 689 cm^-1^ to Fe-OOH stretching vibration, and a newly added peak in NZVI at 1118 cm^-1^ to carbon-O stretching vibration. Notably, the characteristic peaks associated with carboxyl group (COOH), phenolic hydroxyl/hydroxyl group (OH), aldehyde group (CHO), and carbon-carbon triple bond (C≡C) present in HA were not observed in the NZVI@HA material. This absence could be attributed to the physical coverage of HA’s functional groups by NZVI nanoparticles to some extent (Xu et al., 2020).

In **Supplementary Figure S4**, X-ray photoelectron spectroscopy (XPS) was applied to examine the surface elements of the NZVI@HA material, aiming to investigate the composition and valence changes of the elements. **Supplementary Figure S4A** displays the results of the full spectrum examination of NZVI@HA. The characteristic peaks of C1s were observed at 285.36 eV, O1s at 531.37 eV, and Fe2p at 711.34 eV, accounting for 39.26%, 50.64%, and 10.10%, respectively. In **Supplementary Figure S4B**, the fine spectral analysis of element C reveals peaks at 284.87 eV, 286.15 eV, and 288.67 eV corresponding to C-C, C-O, and C=O, with peak areas of 64.87%, 34.36%, and 0.77%, respectively. **Supplementary Figure S4C** displays the fine spectral analysis of element O, showing peaks at 531.23 eV and 529.53 eV corresponding to Fe-O and C-O, with peak areas of 89.44% and 10.56%, respectively. Additionally, **Supplementary Figure S4D** illustrates the fine spectral fitting analysis of Fe2p, where the binding energies of Fe2p1/2 and Fe2p3/2 at 725.56 eV and 712.62 eV represent Fe(III), those at 723.58 eV and 710.47 eV correspond to Fe(II), and the energy at 718.09 eV corresponds to Fe0 (Khalil et al., 2018; Wang et al., 2018; Chen et al., 2023).

In summary, the presence of Fe-O peaks indicates a certain degree of oxidation in NZVI@HA, aligning with findings from various NZVI composites. The appearance of the Fe0 peak signifies the successful loading of NZVI onto the HA surface, consistent with characterization results from scanning electron microscopy (SEM), Fourier transform infrared spectroscopy (FTIR), and X-ray diffraction (XRD), collectively suggesting the effective loading of NZVI on the NZVI@HA surface.

### The impact of HA, NZVI, and NZVI@HA on Cd(II) and Cr(VI) removal from water sources

**Figure 1:**
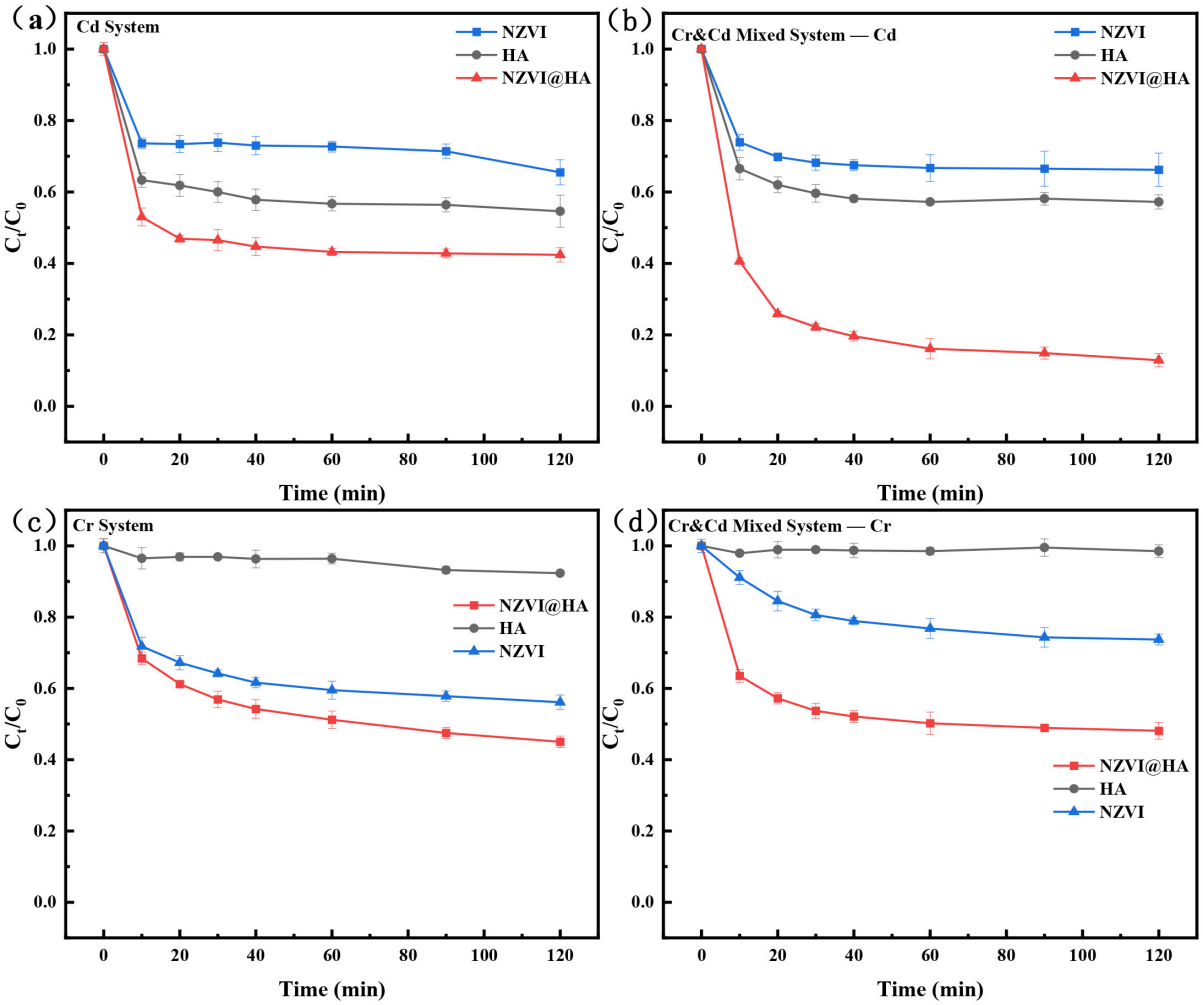
Comparison of Cd(II) Removal using Various Materials (HA, NZVI, NZVI@HA) in Single Cd System **(a)**, Cd(II) Removal in Cr and Cd Mixed System **(b)**, Cr(II) Removal in Single Cr System **(c)**, and Cr(IV) Removal in Cr and Cd Mixed System **(d)**. Vertical bars indicate the standard deviation of the mean (n = 3). Experimental conditions: Initial concentration 50 mg/L, material dosage 1g/L, Temperature (T) = 25°C, pH = 7.

**Figure 1a** illustrates the study on the elimination of Cd(II) from a single Cd system by HA, NZVI, and NZVI@HA. It is evident that the removal efficiencies of the three materials for Cd(II) within 120 minutes were 45.41%, 34.52%, and 57.64%, respectively. The composites exhibited an increase in Cd(II) removal efficiency by 12.20%, with NZVI@HA outperforming both HA and NZVI. This enhancement in Cd(II) removal by NZVI@HA could be ascribed to the negatively charged surface of HA at pH 7 (**Supplementary Figure S5D**), which enhances the adsorption of cationic Cd(II). The negative charge of HA aids NZVI@HA in attracting and immobilizing positively charged Cd(II).

In **Figure 1**, the study on Cr(VI) removal from a single chromium system by HA, NZVI, and NZVI@HA reveals that HA exhibited poor removal efficiency at 7.7%, while NZVI and NZVI@HA achieved removal efficiencies of 43.90% and 55.43%, respectively. The lower removal efficiency of HA can be accredited to the inherent negative charges on the surface of humic acid, which repels Cr(VI). The presence of HA reduces NZVI agglomeration, increases surface adsorption sites, and facilitates electron transfer due to functional groups on the humic acid surface, thereby accelerating Cr(VI) elimination and improving removal rates by NZVI@HA.

**Figures 1b, d** depict the amputation studies of Cr(VI) and Cd(II) in mixed systems of HA, NZVI, and NZVI@HA. Compared to the solo system, the removal efficiency of Cd(II) by NZVI@HA increased by 30.86%, while the elimination efficacy of Cr(VI) remained relatively stable. This enhancement can be attributed to the strong reducing properties of NZVI@HA, which transforms Cr(VI) to Cr(III) and forms insoluble precipitates or complexes with Cd(II) through the oxidation products of NZVI. Additionally, the existence of HA provides additional active sites for NZVI@HA, with functional groups on the HA surface catalyzing reactions, promoting electron transfer, and accelerating the elimination of Cr(VI) and Cd(II).

### The impact of environmental factors on the elimination of Cd(II) and Cr(VI) from water bodies

The pH level is a crucial environmental factor that impacts the amputation efficiency of Cd(II) and Cr(VI) from aqueous systems. It not only influences the chemical state of pollutants but also affects the surface properties and efficacy of removal materials (Zou et al., 2019). The effect of pH on the elimination of Cd(II) and Cr(VI) from a mixed system of Cd and Cr by NZVI@HA is depicted in **Supplementary Figure S5**. The elimination of Cr(IV) by NZVI@HA decreased as ambient pH increased, with the highest removal rate of 85.72% observed at pH=2. This trend can be explained by the predominance of CrO_4_^2-^ and Cd2+ ions in the mixed system under acidic conditions, accounting for 65.24% and 35.67%, respectively (refer to **Supplementary Figure S5C**). A small amount of H_2_CrO_4_(aq) was present at pH < 2, while the presence of HCrO^4-^ decreased and CrO_4_^2-^ ions increased when pH exceeded 6.5 (**Supplementary Figure S5C**). At pH < 7, the positively charged surface of NZVI@HA with a zeta potential of 2.22 mV facilitated the conversion of Cr(IV) to Cr(III) (Zhong et al., 2023), leading to enhanced Cr(VI) amputation by NZVI@HA at low pH levels (Ramos-Hernández et al., 2022).

In **Supplementary Figure S5B**, the impact of pH on the elimination of Cd(II) by NZVI@HA is illustrated. The amputation efficiency of Cd(II) augmented gradually with rising pH, reaching a peak of 94.8% at pH=7. However, at pH=9, the elimination rate of Cd(II) by NZVI@HA reached 94% within 10 minutes, likely due to Cd(II) existing as Cd(OH)_2_ precipitation when pH exceeded 8 (Xie et al., 2020) (**Supplementary Figure S5C**). **Supplementary Figure S5D** displays the zeta potentials of humic acid, NZVI, and NZVI@HA at varying pH levels of 2, 3, 5, 7, and 9. The zeta potentials of humic acid, NZVI, and NZVI@HA exhibited a decreasing trend with increasing pH, turning negative when pH surpassed 7. This indicates that higher pH levels enhance cation removal, while lower pH levels improve anion removal, aligning with the observed effects of pH on Cd(II) and Cr(VI) removal in water systems.

**Supplementary Figures S6A, B** illustrate the impact of initial concentration on the amputation of Cd(II) and Cr(IV) in the mixed system of Cr and Cd by NZVI@HA. It is evident that the confiscation of Cd(II) by NZVI@HA progressively decreased with increasing initial concentrations of Cd and Cr. The removal rate of Cd(II) dropped from 81.7% to 27.2% as the initial concentration of Cr and Cd increased from 30 to 90 mg L^-1^. Similarly, the elimination of Cr(VI) by NZVI@HA decreased from 81.7% to 27.2%. This decline in Cd(II) removal can be ascribed to the saturation of presence of active sites on the surface of NZVI@HA with rising initial concentrations of Cd and Cr. The decline in Cr(VI) amputation was due to a significant portion of active sites being occupied by Cr(VI) at lower concentrations, leading to rapid chemical adsorption. As the Cr(VI) concentration increased, the proportion of adsorption on non-reactive active sites also increased, limiting the Cr(VI) adsorption rate (Stern et al., 2022). Additionally, the thickening of the core-shell structure and the formation of a multilayer shell structure after NZVI@HA corrosion under high Cr(VI) concentrations could slow down the adsorption rate by decreasing the electron transfer rate of NZVI@HA. The electrostatic adsorption between Cr(VI) and the positively charged core-shell structure of NZVI@HA became more pronounced.

In **Supplementary Figure S6c**, the effects of competitive ions Na^+^, Cu^2+^, Zn^2+^, Mg^2+^, Mn^2+^, and NH_4_^+^ on the elimination of Cd(II) from the mixed system of Cr and Cd by NZVI@HA are depicted. All these cations exhibited an blocking impact on Cd(II) elimination by NZVI@HA, with Zn2+ significantly inhibiting the removal rate by 55.3%. The order of inhibition for Cd(II) removal by NZVI@HA was Zn2+ > Cu2+ > Mn2+ > Mg2+ > Na+ > NH4+, suggesting that these competing ions compete with Cd(II) for adsorption sites on the NZVI@HA surface, reducing Cd(II) adsorption opportunities.

The presence of Na^+^, Cu^2+^, Zn^2+^, Mg^2+^, Mn^2+^, and NH_4_^+^ influenced the amputation of Cr(VI) in the mixed system of Cr and Cd by NZVI@HA, with all these cations promoting Cr(VI) removal to some level. Cu^2+^ notably boosted the amputation rate of Cr(VI) in the water column significantly, with a 58.6% increase. The promotion order for Cr(VI) removal by NZVI@HA was Cu^2+^ > NH_4_^+^ > Mn^2+^ > Zn^2+^ > Mg^2+^ > Na^+^. These ions may engage in ion exchange processes with other substances on the NZVI@HA surface or in the medium, indirectly facilitating the adsorption or reduction of Cr. Additionally, some cations, especially Cu^2+^, may act as catalysts due to their strong electron acceptance ability, potentially accelerating the reduction of Cr(VI) (Liu et al., 2020).

To further explore the adsorption effects of NZVI@HA on Cd(II) and Cr(VI) in different treatment systems, primary (**Supplementary Figure S7C**) and secondary (**Supplementary Figure S7D**) kinetic models were proposed and fitted following the method of Xu et al. (2023). The adsorption kinetic parameters of NZVI@HA for various treatment systems are presented in **Supplementary Table S1**. The fitting results indicated that NZVI@HA predominantly relied on chemical adsorption for different treatment systems involving Cd(II) and Cr(VI), as evidenced by the high correlation coefficient values (R22 > 0.999) obtained using the proposed second-level kinetic model compared to the first-level kinetic model.

Additionally, we conducted material recycling and regeneration performance experiments in order to evaluate the economic and environmental sustainability of the materials (**Supplementary Figure S8**), and the results of the study showed that the removal performance of NZVI@HA significantly decreased in the third cycle test, and the removal rates of Cr(VI) and Cd(II) in the 50 mg/L Cr and Cd mixed system decreased to 21.36% and 25.48%, which may be attributed to the fact that during each cycle of use This decline may be attributed to the continuous oxidation of Fe^0^to Fe(II) and Fe(III) during each recycling cycle, and to the deposits formed with Cr(VI) and Cd(II) on the surface of NZVI. These deposits can block the pores of the material, resulting in the loss of active sites and eventual passivation (Wei et al., 2025).

### Mechanisms for Cd(II) and Cr(VI) removal from water by NZVI@HA

**Figure 2:**
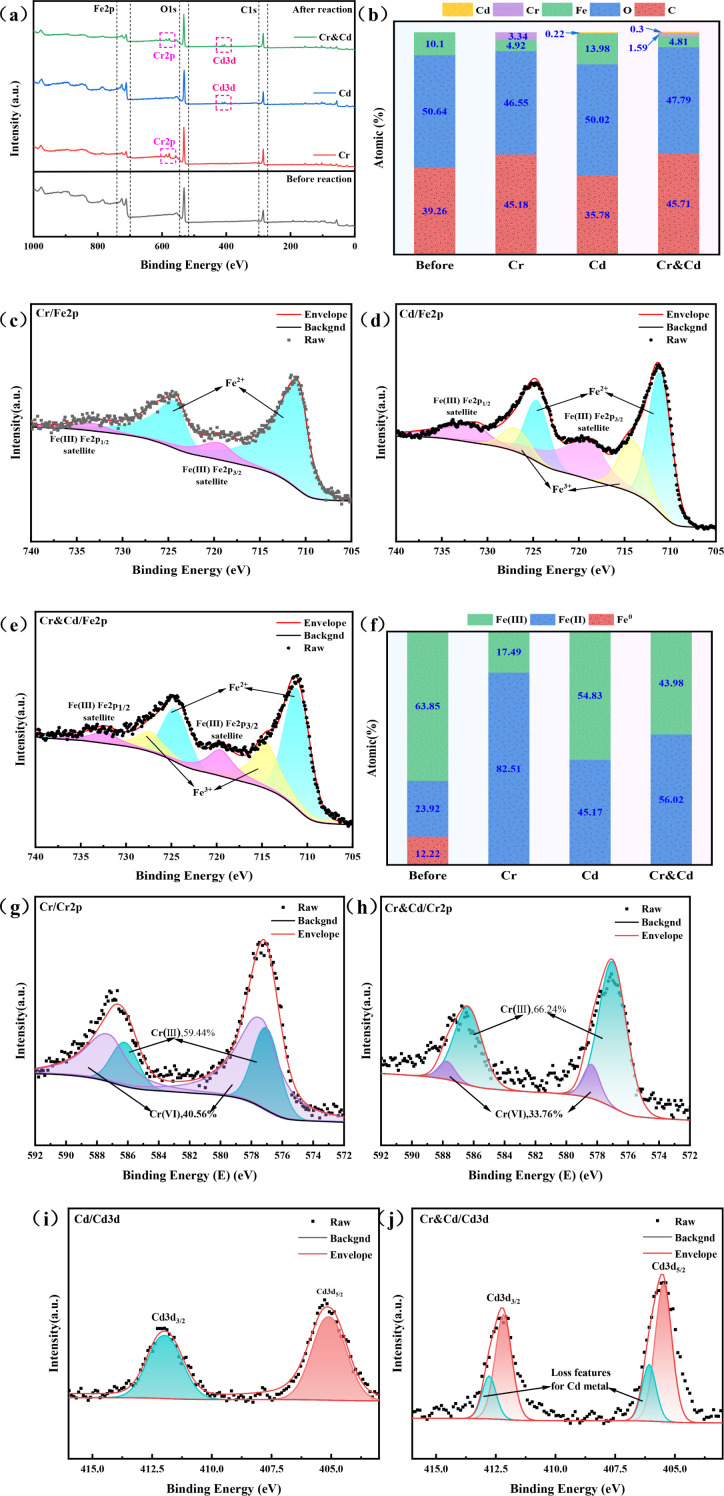
XPS Spectra of NZVI@HA Interacting with Various Systems. Full Spectrum **(a)**, Surface Atomic Changes Pre and Post NZVI@HA Interaction with Different Systems **(b)**, Fe 2p Spectrum during NZVI@HA Reaction with Cr(IV) **(c)**, Fe 2p Spectrum during NZVI@HA Reaction with Cd(II) **(d)**, Fe 2p Spectrum during NZVI@HA Reaction with Cr(IV) and Cd(II) **(e)**, Changes in Fe Content Pre and Post NZVI@HA Interaction with Different Systems **(f)**, Cr 2p Spectrum during NZVI@HA Reaction with Cr(IV) **(g)**, Cr 2p Spectrum during NZVI@HA Reaction with Cr(IV) and Cd(II) **(h)**, Cd 3d Spectrum during NZVI@HA Reaction with Cd(II) **(i)**, Cd 3d Spectrum when NZVI@HA Reacts with Cr(IV) and Cd(II) **(j)**.

In this experiment, the removal rates of NZVI@HA were 57.6% for Cd(II) in a single Cd system and 55% for Cr(VI) in a single Cr system. In a mixed system of Cd and Cr, the removal rates were 87.6% for Cd(II) and 54.3% for Cr(VI. To delve into the removal mechanism of NZVI@HA for Cr(VI) and Cd(II) in the mixed systems, XPS characterization was conducted before and after the reaction (**Figure 2**), aiming to elucidate the removal process of these contaminants in water bodies.

XPS characterization unveiled the elemental variations of NZVI@HA prior and post the reaction with different treatment systems (**Figure 2a**). Following the reaction with single Cr(VI), the elemental content of C enlarged by 5.92%, Fe and O decreased by 4.09% and 5.09%, respectively, while Cr increased by 3.34%. The rise in C content could be linked to the partial consumption of organic matter in HA during Cr(VI) removal, and the decrease in iron and O content indicated the involvement of NZVI@HA in the reduction process of Cr(VI).

After the reaction with single Cd(II), the elemental content of C decreased by 3.48%, Fe increased by 3.97%, O remained stable, and Cd increased by 0.22%. The decline in C content might be due to the creation of a persistent complex between HA and Cd(II), while the upsurge in Fe contents could be attributed to the development of Fe oxides on the NZVI@HA surface, promoting Fe deposition.

Following the mixed reaction with Cr(VI) and Cd(II), elemental carbon content increased by 6.74%, Fe and O decreased by 5.29% and 2.85%, Cr increased by 1.59%, and Cd increased by 0.3%. The elevated Cr and Cd content reflected their accumulation during the reaction process.

**Figures 2c–f** display Fe2p for the reactions of NZVI@HA with Cr(IV) and Cd(II), showcasing changes in Fe content. The Fe0 peak vanished post-reaction, with Fe(II) content increasing by 58.59%, indicating NZVI@HA’s strong reducing properties. The Fe(III) content decreased by 46.36%, possibly due to HA stabilizing Fe(II) and slowing down its oxidation to Fe(III). Compared to the single Cr(IV) reaction, the mixed reaction led to a decline in Fe(II) content and a rise in Fe(III) content.

**Figures 2g, h** illustrate the Cr2p spectra, showing 66.24% Cr(III) and 33.76% Cr(VI) afterward the reaction with Cd(II) and Cr(IV), a 6.8% decrease compared to the single Cr(IV) reaction. Besides, NZVI@HA removes Cr(VI) in Cr single system, 40.56% is adsorbed, 13.08% by reduction to Cr(III), and 46.36% through co-precipitation. In contrast in the mixed chromium and cadmium system, 33.76% of Cr(VI) is removed by adsorption, 43.7% is reduced, and 22.54% is removed through co-precipitation. The presence of Cd(II) likely influenced the active sites on NZVI@HA, enhancing Cr(VI) adsorption capacity or facilitating electron transfer from NZVI to Cr(VI, thereby improving the overall Cr(VI) removal efficiency.

**Figures 2i, j** exhibit Cd3d, revealing new peaks attributed to CdO or Cd(OH)2, indicating the formation of insoluble precipitates or complexes of NZVI@HA with Cd(II). Similarly, NZVI@HA removed 69.73% of Cd(II) through adsorption and 30.27% through co-precipitation in the Cd single system, 48.03% was removed by adsorption and 51.97% by co-precipitation.

Based on the analysis, the removal mechanisms of NZVI@HA are hypothesized as follows: (1) Adsorption: HA’s functional groups provide active sites for direct Cd(II) and Cr(VI) adsorption. (2) Reduction: NZVI@HA acts as a strong reducing agent, altering Cr(VI) to less toxic Cr(III). (3) Co-precipitation: Fe(III) oxidation products form insoluble precipitates with Cr(III) and Cd(II), aiding in their removal from water bodies.

### NZVI@HA synergistic Cr/CdMC for Cd(II) and Cr(VI) elimination

**Figure 3:**
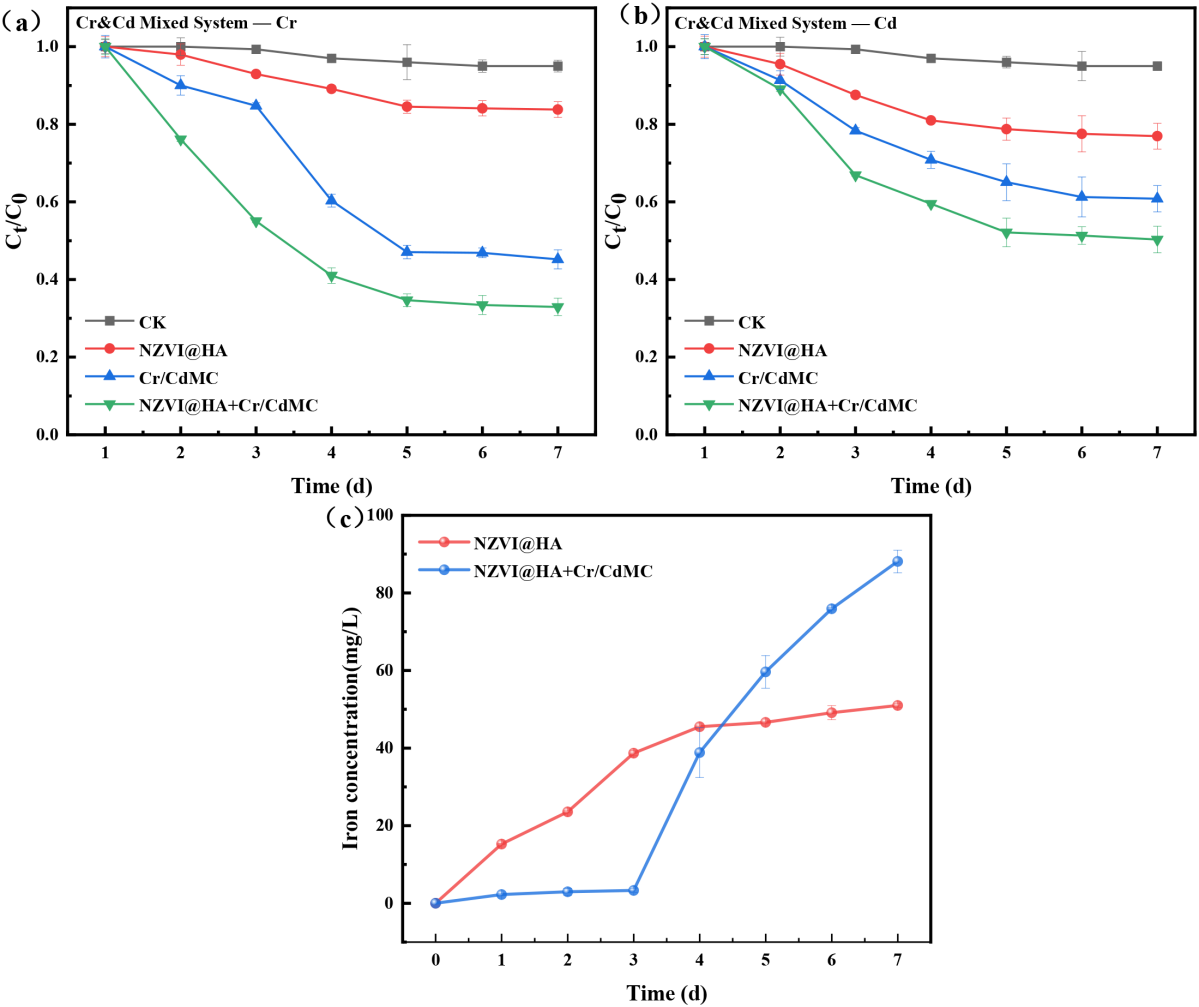
Removal of Cr(IV) **(a)** and Cd(II) **(b)** in Cr&Cd Composite Pollution by NZVI@HA, Cr/CdMC, and NZVI@HA+Cr/CdMC Systems. Dissolved Iron Content after the Reaction of NZVI@HA and NZVI@HA+Cr/CdMC Systems **(c)**. Experimental Conditions: Initial Cr&Cd Concentration is 200 mg/L, Material Dosage is 1 g/L, Cr/CdMC Inoculation Volume is 2% (v/v), Temperature (T) = 25°C, pH = 6.5.

**Figure 3** illustrates the amputation effects of NZVI@HA, Cr/CdMC, and the NZVI@HA+Cr/CdMC systems on Cr(IV) and Cd(II) in a 200 mg L^-1^ Cd and Cr pollution scenario. The results indicate that the NZVI@HA+Cr/CdMC system exhibits the most effective removal of Cd(II) and Cr(IV), with a 12.25% upsurge in removal for both contaminants. Furthermore, it was observed that the amputation rates of Cd(II) and Cr(IV) in the NZVI@HA+Cr/CdMC system increased consistently. In contrast, the Cr/CdMC system showed slower removal rates in the initial 3 days, with a rapid increase on the 4th day, reaching a removal efficiency of 67.06% for Cr(IV) and 49.70% for Cd(II) by the end of the incubation period. This difference may be attributed to NZVI@HA mitigating the impacts of Cd(II) and Cr(IV) stresses on the chromium and cadmium symbiotic flora, while also providing a carbon source to support the symbiotic flora.

In **Figure 3c**, the dissolved iron contents of the NZVI@HA and NZVI@HA+Cr/CdMC systems post-reaction are depicted. It was noticed that the solubilized Fe content in the NZVI@HA+Cr/CdMC system was significantly greater than that in the NZVI@HA system. This suggests that Cr/CdMC accelerated the dissolution of Fe (II) and Fe (III) on the NZVI@HA surface, enhancing the stability of the “core-shell” structure of NZVI@HA. The Fe0 within the inner “core-shell” layer can be removed through reduction, complexation, and co-precipitation with Cr(IV) and Cd(II) (Di et al., 2023).

### Influence and effect of Cr/CdMC on Cd(II) and Cr(VI) amputation by NZVI@HA

**Figure 4:**
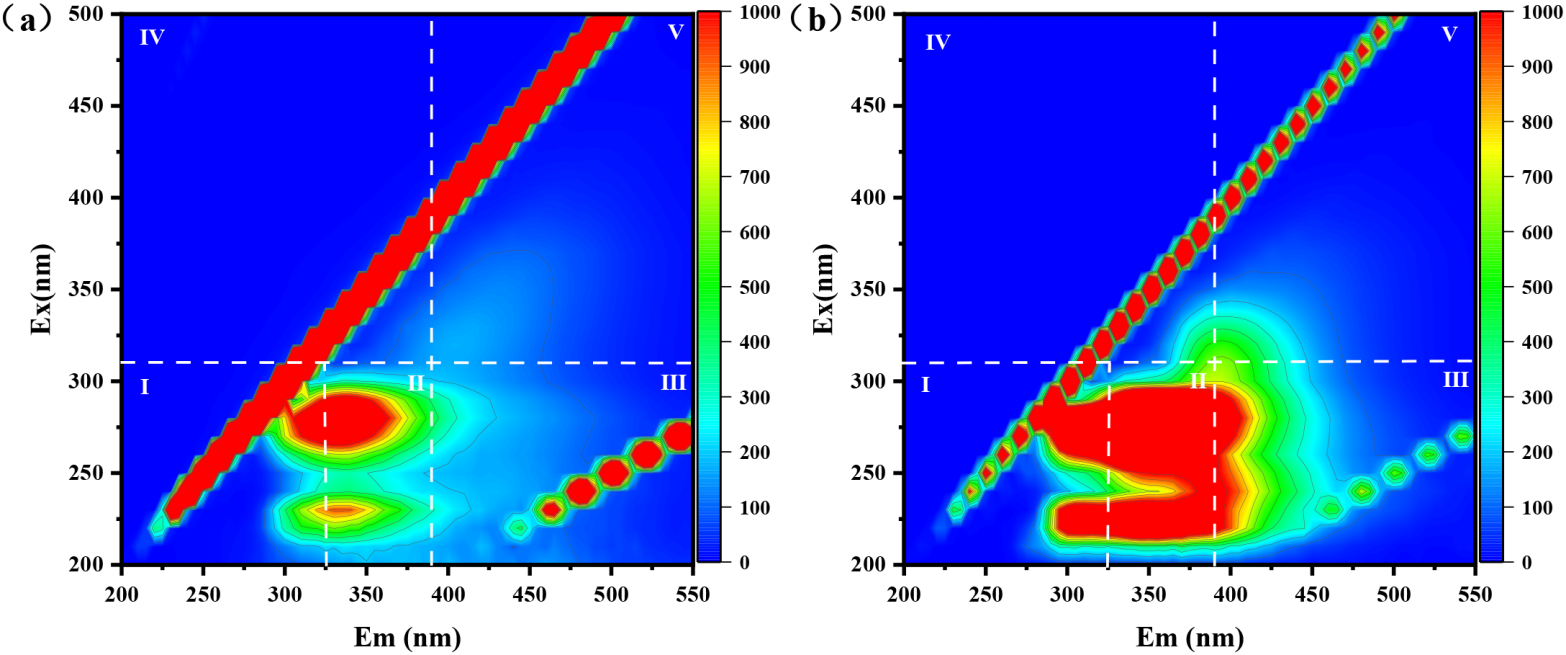
Three-Dimensional Excitation-Emission Matrix (3D-EEM) Fluorescence Spectra of EPS in Cr/CdMC **(a)** and NZVI@HA+Cr/CdMC **(b)** Systems after 7 Days of Reaction.

EPS can serve as a complexing and reducing agent for the amputation of heavy metals from diverse wastewaters (Siddharth et al., 2021). To delve into the adsorption mechanism of extracellular polymers (EPS) on Cd(II) and Cr(VI), 3D-EEM fluorescence spectroscopy was conducted on the Cr/CdMC and NZVI@HA+Cr/CdMC systems (**Figure 4**).

In **Figure 4a**, after 7 days of reaction, the Cr/CdMC system exhibited robust fluorescence intensity in areas I, II, III, IV, and V, indicating the production of tyrosine, tryptophan, fulvic acid substances, protein metabolites, and humic acid substances by the chromium and cadmium symbiotic flora.

In comparison to the Cr/CdMC system, **Figure 4b** shows an increased fluorescence intensity in areas I and II in the NZVI@HA+Cr/CdMC system. This suggests that NZVI@HA enhances the production of tyrosine and tryptophan by the chromium and cadmium symbiotic colonies. The side chains of tyrosine and tryptophan contain -OH and π-π bonds (Nascimento et al., 2019), with tyrosine acting as a reductant to convert Cr(VI) to Cr(III), and tryptophan forming stable complexes with Cr(VI) and Cd(II) through van der Waals force interactions (Tan et al., 2023). The synergistic interaction of NZVI@HA with the chromium and cadmium commensal colonies enhances the amputation of Cd(II) and Cr(VI), aligning with previous experimental results where NZVI@HA+Cr/CdMC increased the amputation of Cd(II) and Cr(VI) by 12.25% and 10.52%, respectively. This leads to the hypothesis that EPS can function as a complexing and reducing agent to eliminate Cd(II) and Cr(VI) from water bodies.

### Effect and influence of NZVI@HA on chromium and cadmium symbiotic flora (Cr/CdMC)

**Figure 5:**
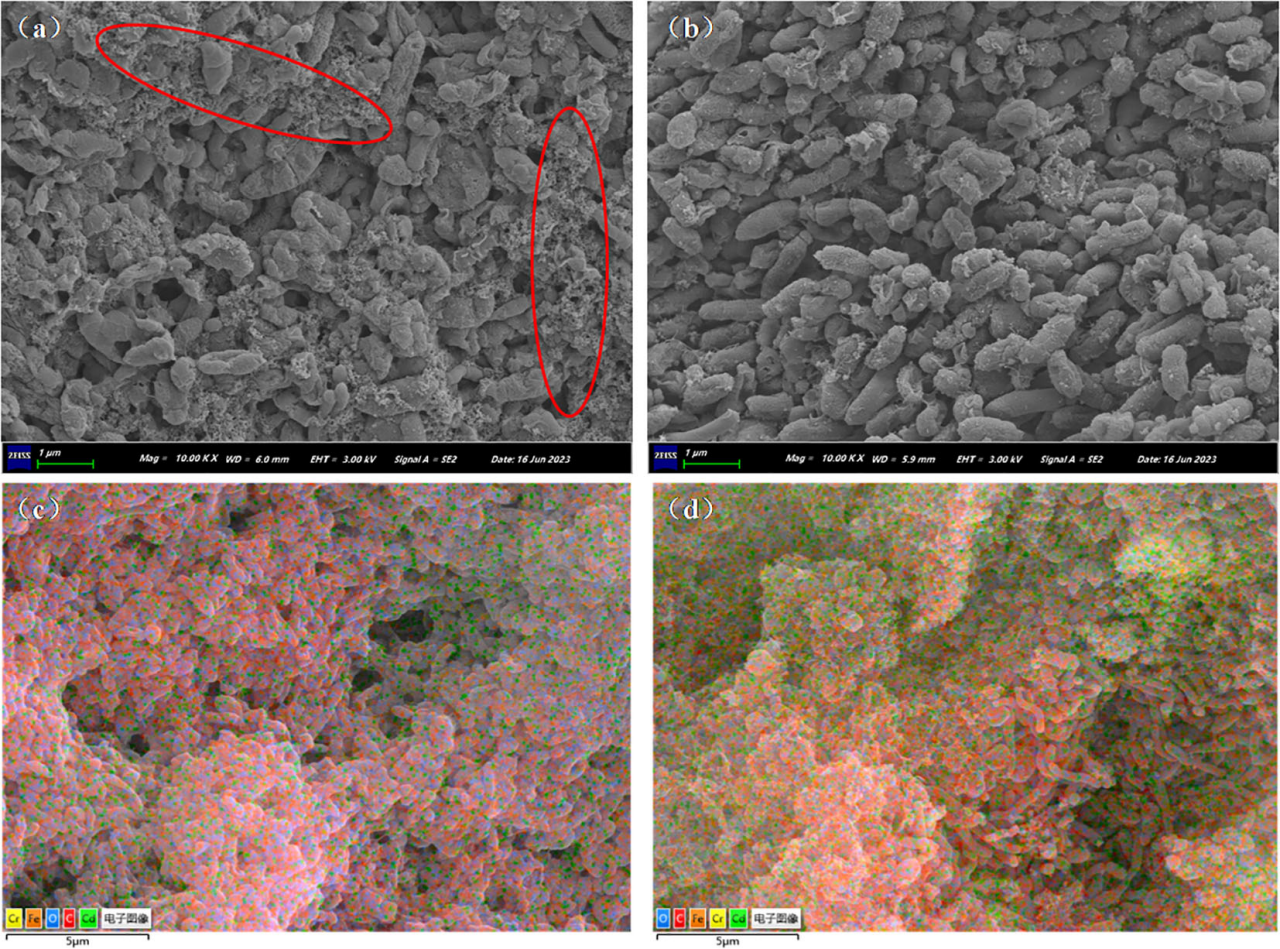
Biological Scanning Electron Microscope Images of Cr/CdMC **(a)** and NZVI@HA+Cr/CdMC **(b)** Systems after Reaction, along with Energy-Dispersive X-ray Spectroscopy (EDS) Layered Images of Cr/CdMC **(c)** and NZVI@HA+Cr/CdMC **(d)** Systems.

**Figure 5** displays the microbial SEM images and EDS layered images of the Cr/CdMC and NZVI@HA+Cr/CdMC systems post-reaction. In **Figure 5a**, the microbial surface of the Cr/CdMC system exhibited tiny pinholes, with some microbes appearing powdered, indicating stress from Cd(II) and Cr(VI). Upon the addition of NZVI@HA, the microbial surface became smoother, with reduced pinholes, suggesting that NZVI@HA alleviated the Cd(II) and Cr(VI) stress on Cr/CdMC and fostered microbial growth. Furthermore, as depicted in **Figures 5c, d**, the elemental carbon in the NZVI@HA+Cr/CdMC system notably declined, while the levels of chromium and cadmium significantly increased. The EDS energy spectrum analysis revealed that in the Cr/CdMC system, carbon, oxygen, chromium, and cadmium accounted for 73.39%, 25.12%, 1.27%, and 0.22%, respectively. In contrast, in the NZVI@HA+Cr/CdMC system, carbon, oxygen, iron, chromium, and cadmium elements constituted 58.23%, 25.92%, 6.96%, 3.31%, and 5.58%, respectively, with a 15.16% reduction in carbon. This reduction suggests the involvement of carbon in the amputation of Cd(II) and Cr(VI) by the NZVI@HA+Cr/CdMC system, possibly due to microorganisms utilizing carbon sources for growth and metabolism (Brown et al., 2022). The increase in iron content indicates partial dissolution of iron by NZVI@HA during the reaction, while the elevated levels of chromium and cadmium elements suggest enhanced removal efficiency of Cr(VI) and Cd(II) by the NZVI@HA+Cr/CdMC system.

**Figure 6:**
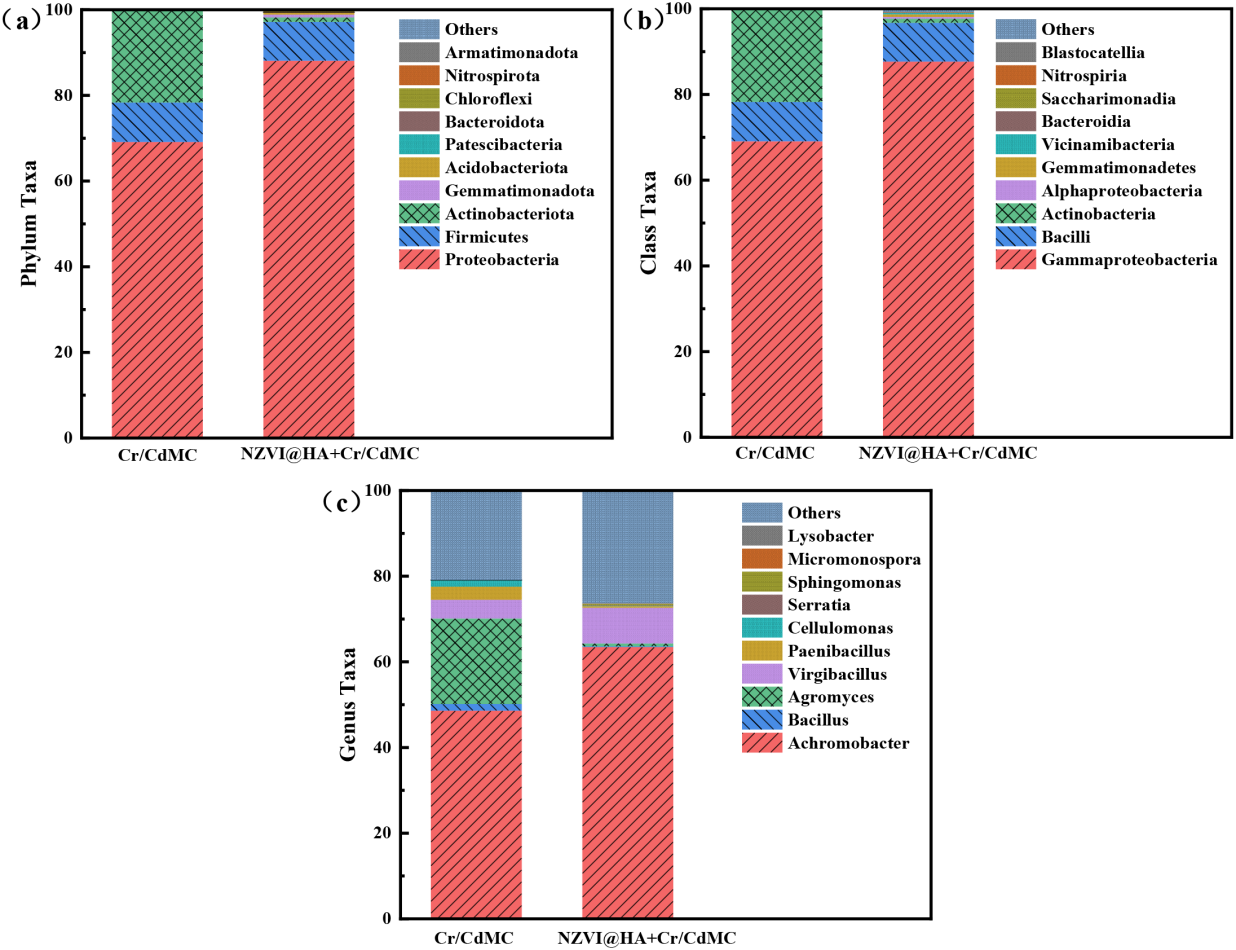
Comparative Analysis of Microbial Community Composition at the Phylum **(a)**, Class **(b)**, and Genus **(c)** Levels in Cr/CdMC and NZVI@HA+Cr/CdMC Systems.

Microbial diversity can assess the ecotoxicity of NZVI@HA on ecosystems and reveal synergistic mechanisms. **Figure 6** illustrates the relative abundance of Cr and Cd symbiotic flora at the “phylum,” “order,” and “genus” levels in the Cr/CdMC and NZVI@HA+Cr/CdMC systems. Proteobacteria, Firmicutes, and Actinobacteriota were the dominant phyla in both systems, with Proteobacteria, Firmicutes, and Actinobacteriota being more prevalent in the NZVI@HA+Cr/CdMC system compared to Cr/CdMC. The relative abundance of Proteobacteria increased by 18.99%, while Actinobacteriota decreased by 20.46% in the NZVI@HA+Cr/CdMC system, indicating a shift in the microbial ecological balance due to the introduction of NZVI@HA. The rise in Proteobacteria abundance suggests its significant role in the microbial ecological balance and removal of Cd(II) and Cr(VI) in the NZVI@HA+Cr/CdMC system (Yu et al., 2021).

At the “genus” level, Achromobacter and Virgibacillus were the dominant genera shared by both systems. The addition of NZVI@HA led to the emergence of new genera such as Bacillus, Agromyces, Paenibacillus-like, and Cellulomonas in the Cr/CdMC system (Zhao et al., 2021). Bacillus and Agromyces belong to the phylum Thick-walled Bacteria, while Agromyces and Cellulomonas are part of the Actinomycetes phylum, indicating some inhibitory effects on specific microorganisms by NZVI@HA.

The Chao1, Faith_pd, and Observed species indices in the NZVI@HA+Cr/CdMC system were markedly greater than those in the Cr/CdMC system (p ≤ 0.05), suggesting that NZVI@HA increased the richness and evolutionary diversity of chromium and cadmium symbiotic flora. The Pielou_e index was slightly lower in the NZVI@HA+Cr/CdMC system, indicating uneven growth of the symbiotic flora, possibly due to their proliferation in the pore spaces of NZVI@HA. The Shannon index was also slightly lower, supporting the idea that NZVI@HA reduced environmental stresses on the symbiotic flora and promoted microbial diversity.

In summary, NZVI@HA acts as a potent reducing agent directly involved in heavy metal removal. It alleviates stress on Cr and Cd symbiotic flora, enhances their activity and metabolism, and indirectly boosts the removal of Cd(II) and Cr(VI).

### Impact of NZVI@HA on the functional genes of chromium and cadmium symbiotic bacterial colonies

**Figure 7:**
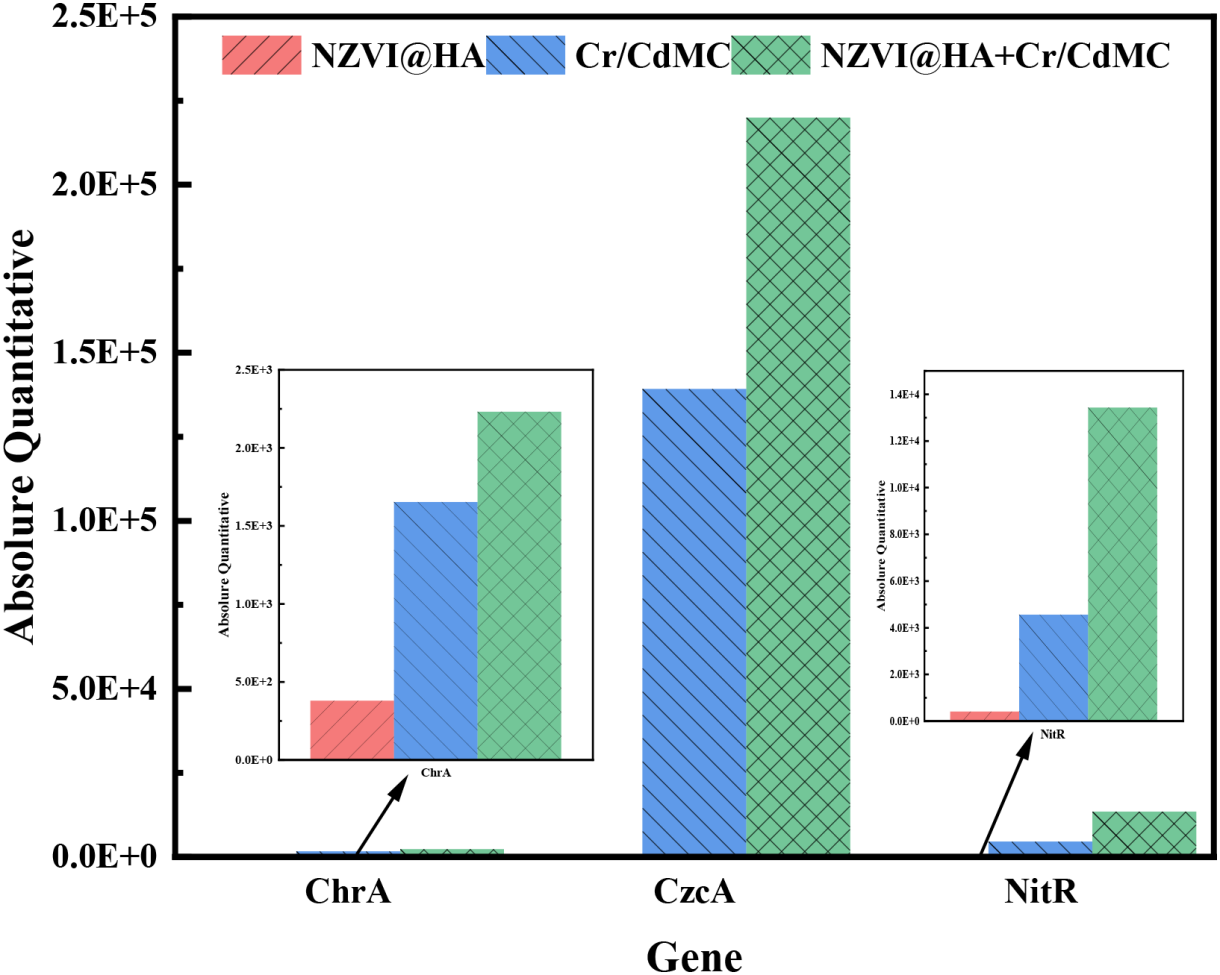
Quantification of chrA, czcA, and nitR Functional Gene Expression Levels in NZVI@HA, Cr/CdMC, and NZVI@HA+Cr/CdMC Systems Post-Culture.

**Figure 7** illustrates the expression levels of the chrA, czcA, and nitR functional genes in the NZVI@HA, Cr/CdMC, and NZVI@HA+Cr/CdMC systems, analyzed through fluorescence quantitative PCR in the reaction solution after 7 days of incubation. In the respective systems, the gene abundance of the Cr resistance-related gene chrA was 379.324, 1652.82, and 2231.60 (gene copy number/bacterial solution mL), the gene abundance of the Cd resistance-related gene czcA was 106.86, 139,178.78, and 21,992,222.12, and the gene abundance of the Cr reduction-related gene nitR was 395.36, 4556.58, and 13425.15. Overall, the gene abundance of chrA, czcA, and nitR functional genes increased with the addition of NZVI@HA, indicating that NZVI@HA promoted the expression of specific functional genes such as chrA, czcA, and nitR.

Furthermore, previous experiments confirmed that the confiscation of Cr(IV) from Cr and Cd pollution was 15.8% greater in the NZVI@HA+Cr/CdMC system than in the Cr/CdMC system on day 7. Additionally, the copy number of chromium-reducing gene *nitR* was 66.06% higher in the NZVI@HA+Cr/CdMC system compared to the Cr/CdMC system. This outcome validates the role of NZVI@HA in enhancing the expression of Cr and Cd-related reduction genes in the symbiotic flora of Cr and Cd, align with the findings of Tan et al. Moreover, the increased gene expression of the chromium reduction-related gene *nitR* may be a key factor contributing to the more efficient amputation of Cr(VI).

## Conclusion

The NZVI@HA has the capability to reduce Cr(VI) to the less toxic Cr(III), while Fe0 undergoes oxidation to Fe(II) and Fe(III). The oxidation products of Fe0, Fe(II), and Fe(III) can react with Cr(III) and Cd(II) to form compounds like Fe(OH)3-Cd(OH)2 and Fe(OH)_3_-Cr(OH)_3_, facilitating the elimination of Cd(II) and Cr(VI) from water. Additionally, the NZVI@HA+Cr/CdMC system enhanced the amputation of Cd(II) and Cr(IV) by 10.52% and 12.25%, respectively. Results from 3D-EEM analysis revealed that EPS may serve as a reductant and complexing agent for heavy metal removal from diverse effluents. Compounds like tyrosine may reduce Cr(VI) to Cr(III) with reduced toxicity, while tryptophan can form stable complexes with Cr(VI) and Cd(II) through van der Waals force interactions. Moreover, NZVI@HA can alleviate the stress on chromium and cadmium symbiotic bacterial colonies (Cr/CdMC) caused by Cd(II) and Cr(VI), promoting their activity, metabolism, and indirectly aiding in the removal of Cd(II) and Cr(VI). In conclusion, the composite material NZVI@HA, synthesized by modifying NZVI with humic acid, proves to be a promising solution for alleviating Cd and Cr pollution from the environment.

The NZVI@HA composite, utilizing natural humic acid (HA) as a carrier, avoids secondary pollution risks through its biodegradability, aligning with green and sustainable remediation principles. Its high efficiency significantly reduces the dosage of remediation agents and operational costs. For instance, under an optimized mass ratio (HA: Fe(II) = 3:1), the removal efficiencies for Cr(VI) and Cd(II) reach 38.3% and 85.6%, respectively. The selective response to competitive ions (e.g., Cu^2+^ enhances Cr removal, while Zn^2+^ inhibits Cd removal) provides precise regulatory guidance for targeted heavy metal removal in complex contaminated systems, preventing resource waste and over-remediation. Furthermore, NZVI@HA enhances microbial tolerance to heavy metal stress by activating the metabolic activity and upregulating reduction-related gene expression in chromium-cadmium co-contaminated microbial consortia (Cr/CdMC), indirectly improving pollutant removal efficiency. This mechanism supports *in-situ* bioaugmentation at contaminated sites, facilitating the self-restoration of damaged ecosystems.

Compared with the existing studies (**Table 1**), this study was carried out under neutral (pH=7) conditions, which is more economical and practical. Notably, the removal of Cr(IV) and Cd(II) by NZVI@HA in the same system reached 55.46% and 96.24, respectively, rare among the existing studies. Moreover, we achieved microbial synergistic removal of Cr(IV) and Cd(II) at 200 mg/L with a removal rate of 70%, which provides a novel approach for treating high concentration heavy metal pollution.

The elucidated interfacial reaction mechanisms—such as Cr(VI) reduction to less toxic Cr(III), Fe(OH)_3_-Cr/Cd co-precipitation, and extracellular polymeric substance (EPS)-mediated complexation, provide a theoretical foundation for optimizing critical parameters (e.g., pH adjustment, material ratios) in practical remediation processes. Additionally, the scalable preparation feasibility and stability of NZVI@HA under dynamic environmental conditions enable its engineering applications, such as serving as permeable reactive barrier (PRB) fillers or soil stabilizing agents. This advancement promotes the transition of heavy metal remediation technologies from laboratory-scale research to field implementation, offering environmental management authorities an integrated solution characterized by high efficiency, cost-effectiveness, and ecological compatibility.

It serves as an efficient and environmentally friendly remediation material, offering a valuable strategy for addressing composite pollution in environmental settings.

## Data Availability

All relevant data is contained within the article. The original contributions presented in the study are included in the article/**Supplementary Material**, further inquiries can be directed to the corresponding author/s.
