# Supplementary material for: Microbial removal mechanism of chromium and cadmium by humic acid-loaded nano zero-valent iron prepared by liquid-phase reduction method
Source: Front Plant Sci. 2025 Aug 5;16:1596063. doi: 10.3389/fpls.2025.1596063 (PMC12392117; doi:10.3389/fpls.2025.1596063)
Supplement: Supplementary file 1 [file DataSheet1.pdf]

Text S1

Preparation of catalysts

The nZVI was prepared by the liquid-phase reduction method. $NaBH_4$ aqueous solution was added to the $FeSO_4 \cdot 7H_2O$ solution, and Fe^0.

nZVI was obtained through redox reaction. The specific preparation process is as follows:

① Preparation of deoxygenated ultrapure water and deoxygenated anhydrous ethanol: Replace the blue-mouth bottle with a stainless steel flow phase replenishment cover, and connect the rubber tube and N_2 bottle. Control the pressure of the N_2 outlet, and keep it for 30 minutes to deoxygenate the ultrapure water and anhydrous ethanol.

② Preparation of ice water for $NaBH_4$ solution: Take an appropriate amount of the prepared deoxygenated ultrapure water in the refrigerator for 1-2 hours, and adjust its pH to 10-11 before use to prevent the hydrolysis of $NaBH_4$.

③ Preparation of $FeSO_4 \cdot 7H_2O$ solution: Weigh 1.3908 g of $FeSO_4 \cdot 7H_2O$ into a 250 ml flat-bottom three-necked flask, add 100 ml of deoxygenated ultrapure water, introduce high-purity N_2 into the three-necked flask, and stir and dissolve it by a mechanical stirrer. The concentration of $FeSO_4 \cdot 7H_2O$ obtained is 0.05 mol/L. After dissolution, continue to agitate and bubble for 15 minutes.

④ Preparation of NaBH4 solution: Weigh 0.3783 g of $NaBH_4$ and make up to 50 ml with the prepared ice water. The concentration of the obtained $NaBH_4$ solution is 0.2 mol/L. The solution should be prepared and used immediately.

⑤ Add the NaBH4 solution to the three-necked flask through a constant pressure dropwise funnel at a speed of 1-2 drops/second, and agitate and bubble continuously. Black particles appear in the flask, and finally it becomes a black suspension. After the $NaBH_4$ solution is added completely, agitate and bubble continuously for 15 minutes to ensure complete reaction.

⑥ Use a magnetic separator to separate the prepared nZVI and the solvent, and then wash it three times with deoxygenated anhydrous ethanol and ultrapure water respectively to prevent nZVI oxidation and remove the remaining borohydride.

⑦ Finally, vacuum dry at 60 ℃ for 12 hours and store for future use.

The preparation of humic acid-loaded nano zero-valent iron (NZVI@HA) was also carried out by the liquid-phase reduction method. Purified HA and ferrous sulfate heptahydrate ($FeSO_4 \cdot 7H_2O$) were added to a wide-mouth bottle in different ratios (HA:Fe(II) = 1:2, 1:1, 2:1, 3:1, 4:1), followed by the addition of 100 mL of deoxygenated pure water. The wide-mouth bottle was placed on a shaker at 150 rpm for 12 hours to ensure the thorough mixing of HA and $FeSO_4$·7H2O. Then, under the experimental condition of nitrogen gas flow, the mixture was transferred to a three-necked flask, and sodium borohydride solution prepared with cold water was added dropwise using a separatory funnel. After complete addition, the mixture was stirred for another 10 minutes under a nitrogen atmosphere to ensure complete reduction of Fe(II) and Fe(III) to Fe0. The reaction resulted in a solution with black precipitate. The three-necked flask was sealed and protected by nitrogen gas. The black precipitate was separated by magnetic adsorption, and the supernatant was discarded. The precipitate was washed three times with cold water and three times with anhydrous ethanol, and finally, NZVI@HA with different ratios was obtained.

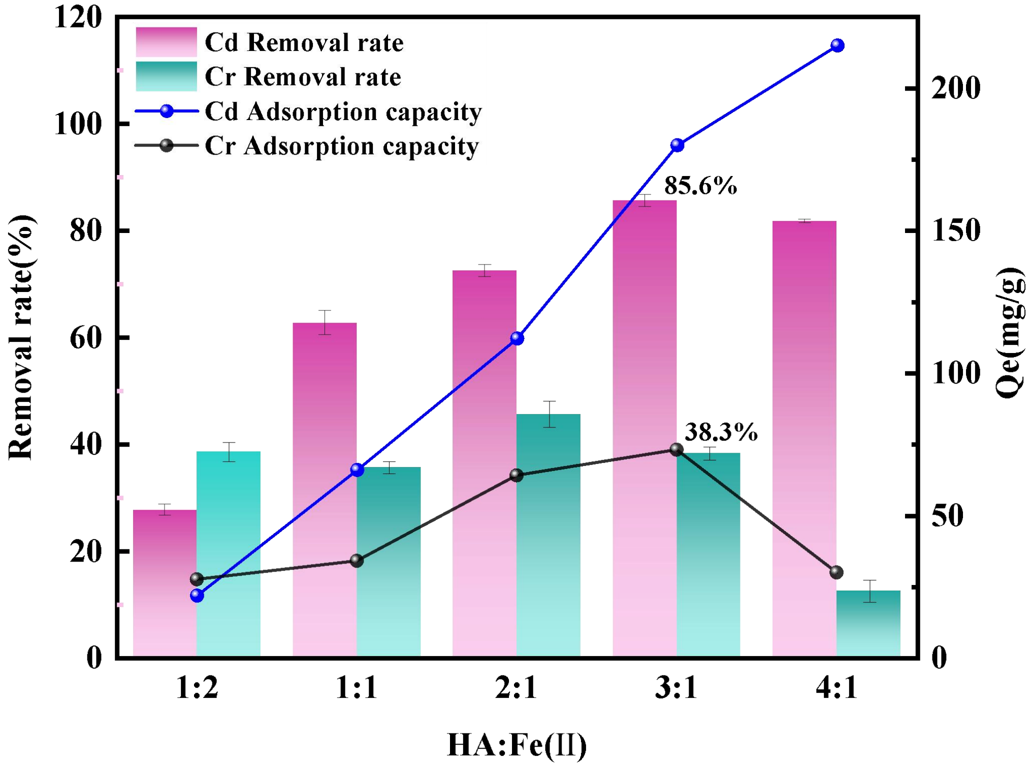

Figure S1. Removal efficiency and adsorption capacity of Cr(VI) and Cd(II) by Fe(II) per unit mass of NZVI@HA synthesized with varying HA to Fe(II) ratios. Error bars indicate the standard deviation of the mean (n = 3). Experimental conditions: initial concentration of 50 mg/L, NZVI@HA dosage of 1 g/L, temperature of 25°C, and pH of 7.

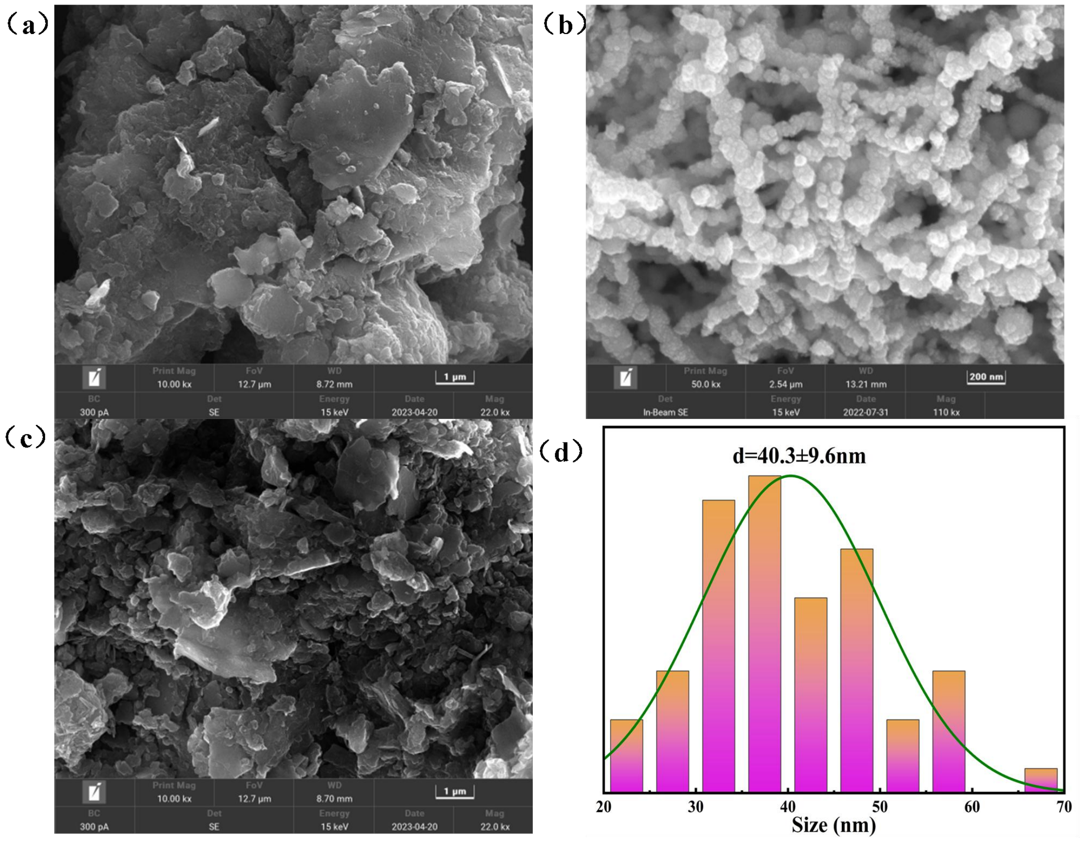

Figure S2. Scanning Electron Microscopy (SEM) images of (a) Humic Acid (HA), (b) Nanoscale Zero-Valent Iron (NZVI), and (c) NZVI coated with Humic Acid (NZVI@HA), along with the corresponding particle size distribution.

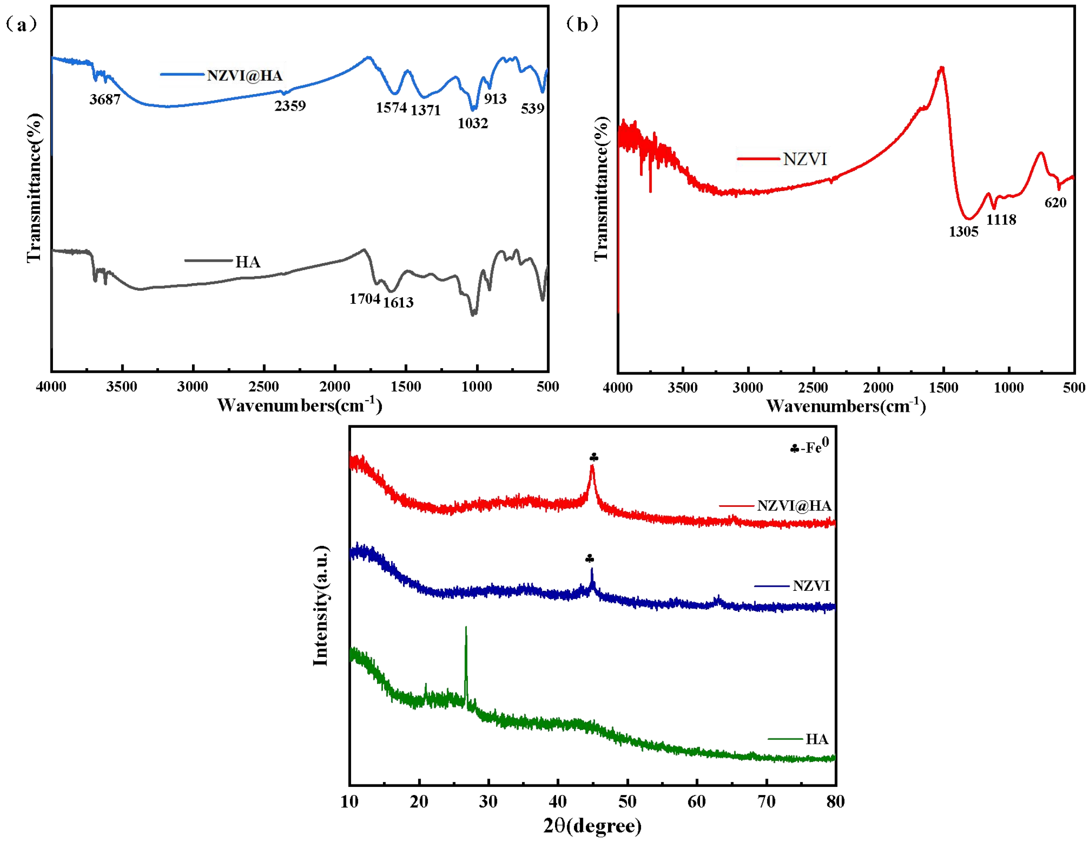

Figure S3. (a) FTIR spectra of Humic Acid (HA) and NZVI coated with Humic Acid (NZVI@HA), (b) FTIR spectra of Nanoscale Zero-Valent Iron (NZVI), and (c) XRD spectra of HA, NZVI, and NZVI@HA.

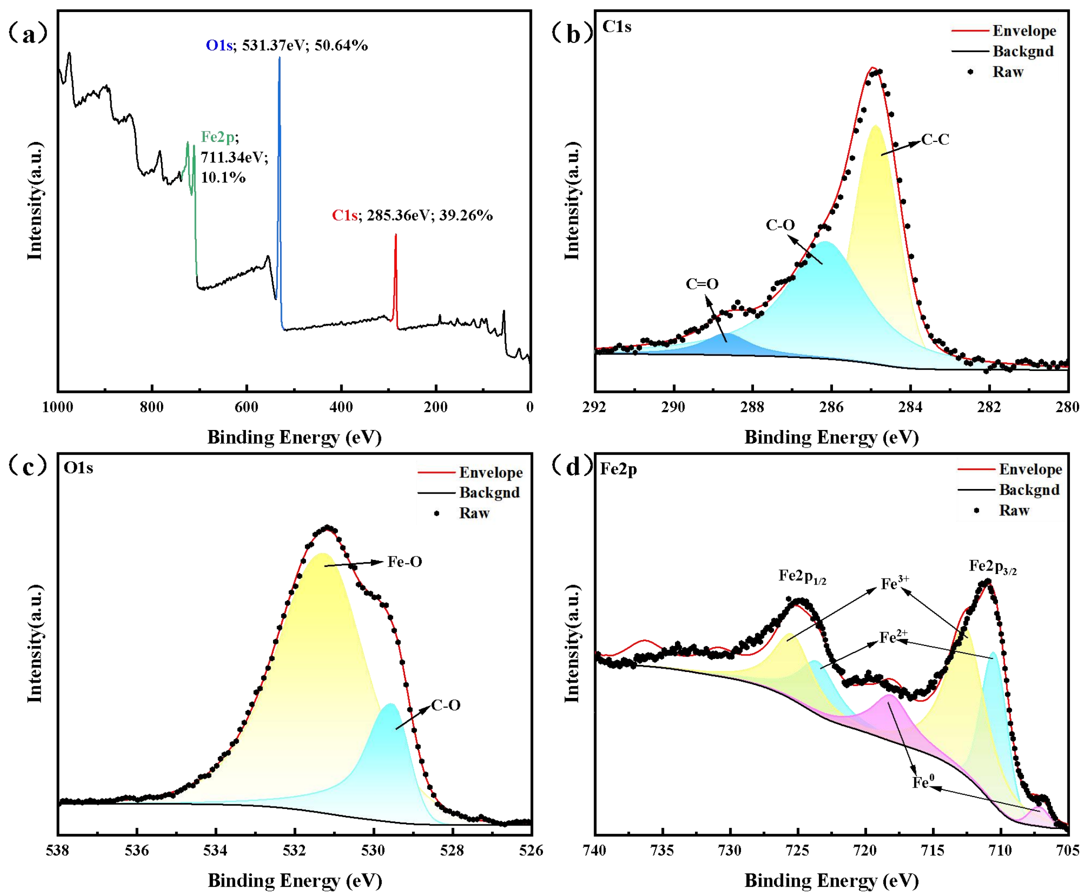

Figure S4. XPS spectra of NZVI coated with Humic Acid (NZVI@HA): (a) Full spectrum, (b) C1s binding energy region, (c) O1s binding energy region, and (d) Fe2p binding energy region.

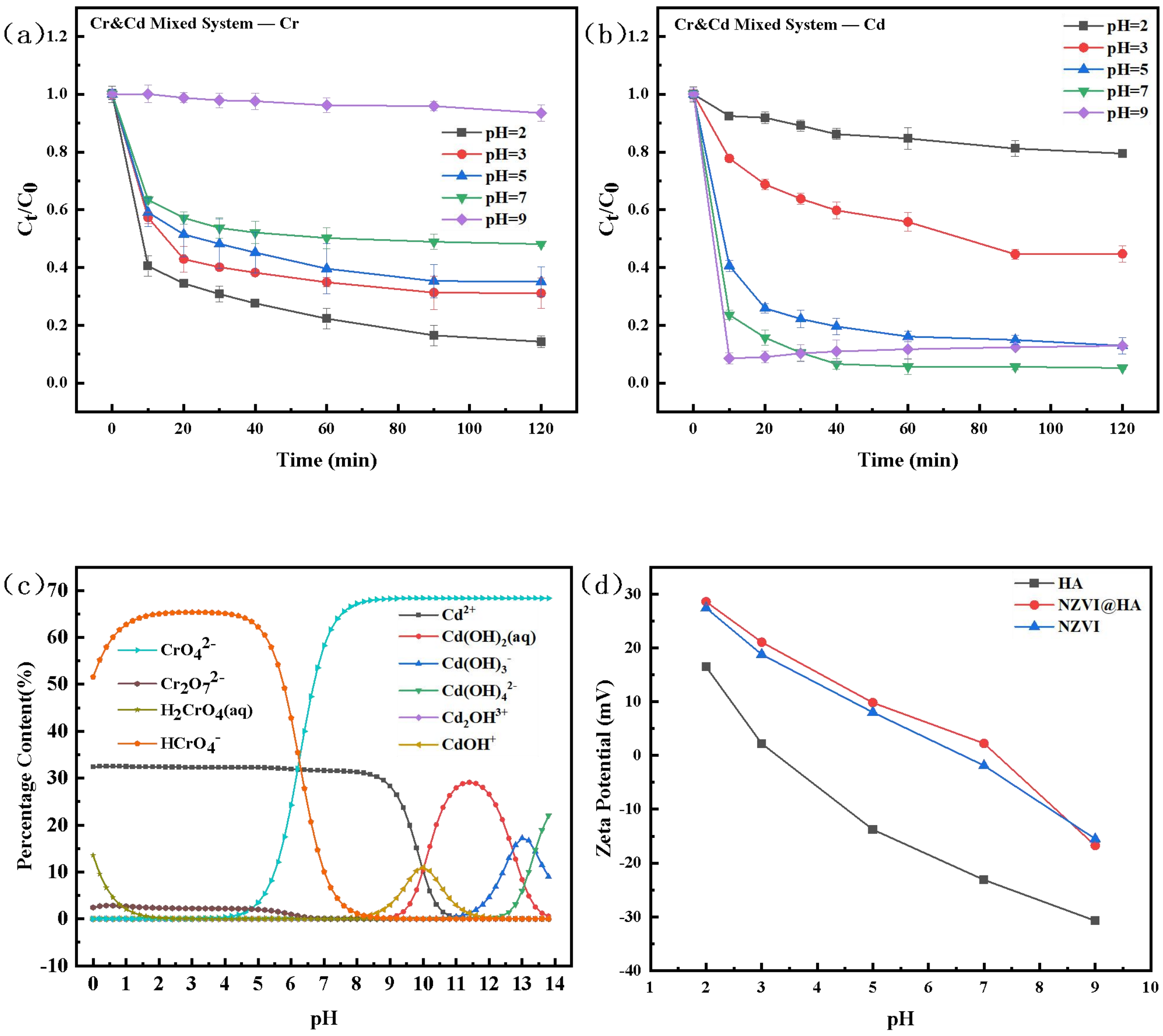

Figure S5. Effect of pH on the removal of Cr(IV) (a) and Cd(II) (b) from a Cr and Cd mixed system by NZVI@HA. Vertical bars represent the standard deviation of the mean value (n = 3). Experimental conditions: initial concentration of 50 mg/L, NZVI@HA dosage of 1 g/L, temperature of 25 ℃, and pH of 7. (c) Simulation of ion percentage content in the Cr and Cd mixed system using Visual MINTEQ. (d) Zeta potential of HA, NZVI, and NZVI@HA at different pH levels.

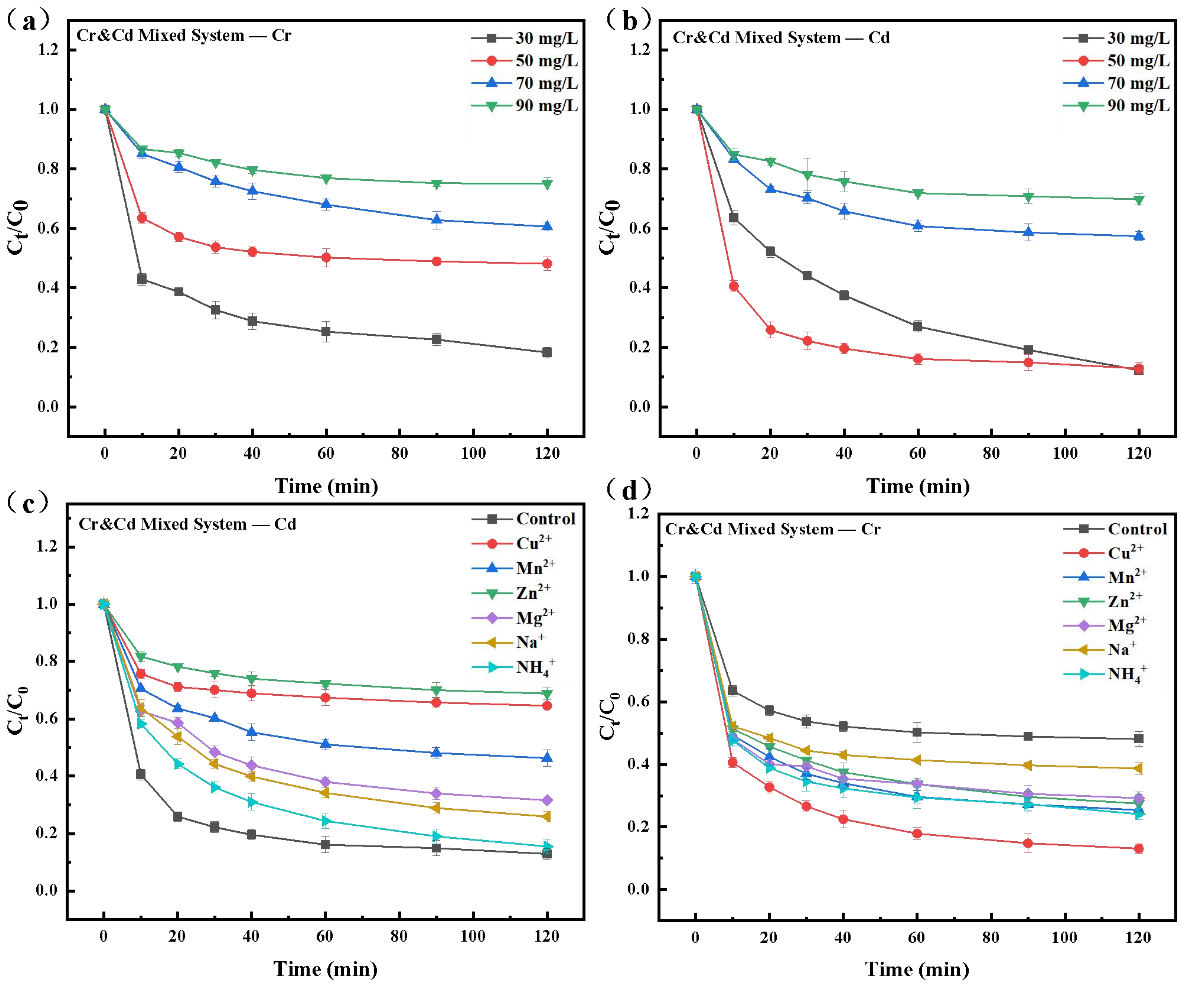

Figure S6. Effect of initial concentration on the removal of Cr(IV) (a) and Cd(II) (b) by NZVI@HA from a mixed Cr and Cd system. Effect of competitive ions on the removal of Cd(II) (c) and Cr(IV) (d) from the mixed system by NZVI@HA. Vertical bars represent the standard deviation of the mean value (n = 3). Experimental conditions: initial concentration of 50 mg/L, NZVI@HA dosage of 1 g/L, temperature of 25 ℃, and pH of 7.

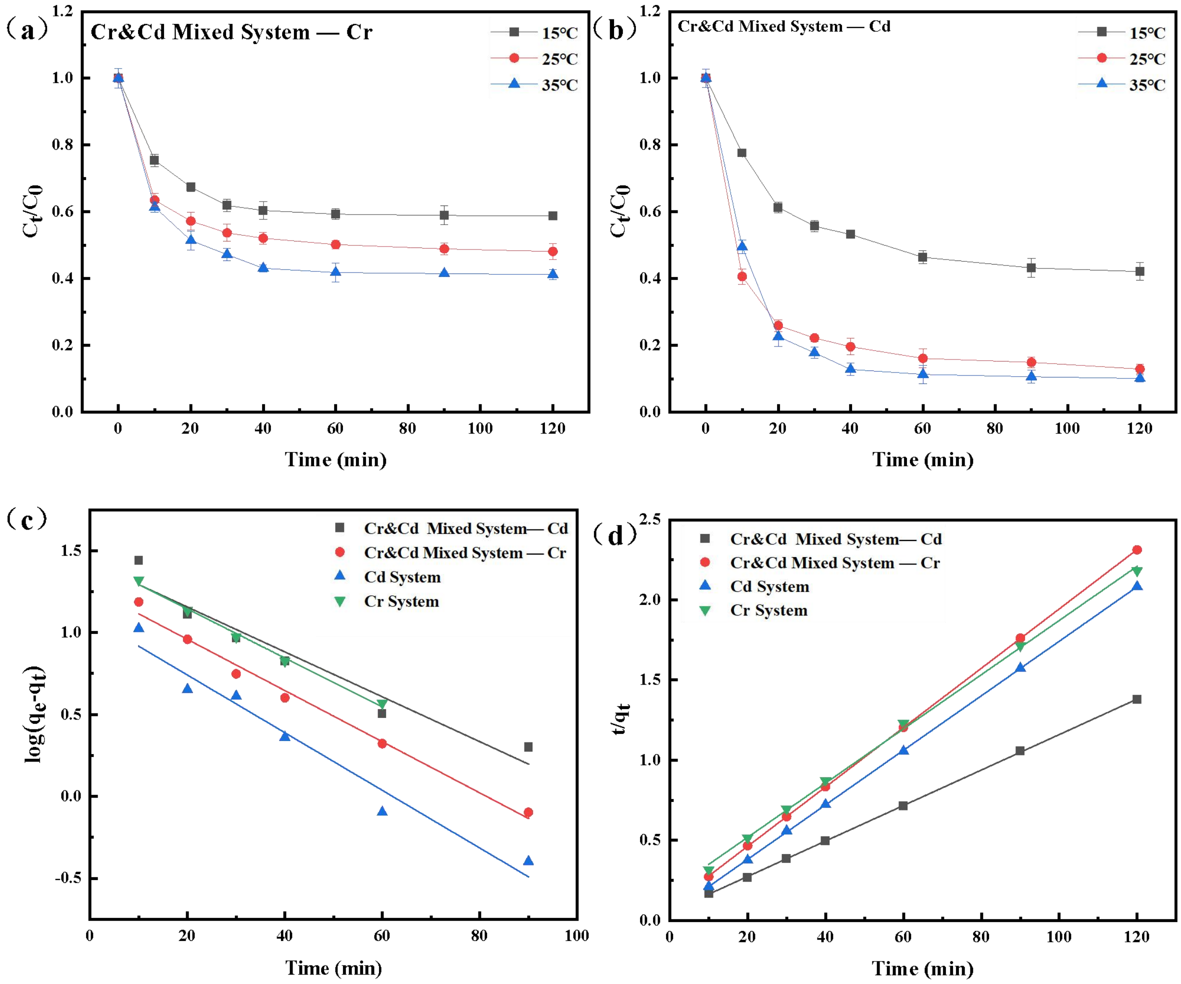

Figure S7. Effect of competitive ions on the removal of Cr(IV) (a) and Cd(II) (b) from Cr and Cd mixed systems by NZVI@HA. Vertical bars represent the standard deviation of the mean value (n = 3). Experimental conditions: initial concentration of 50 mg/L, NZVI@HA dosage of 1 g/L, temperature of 25 ℃, and pH of 7. Comparison of NZVI@HA performance with pseudo-first-order (c) and pseudo-second-order (d) kinetic models for different treatment systems.

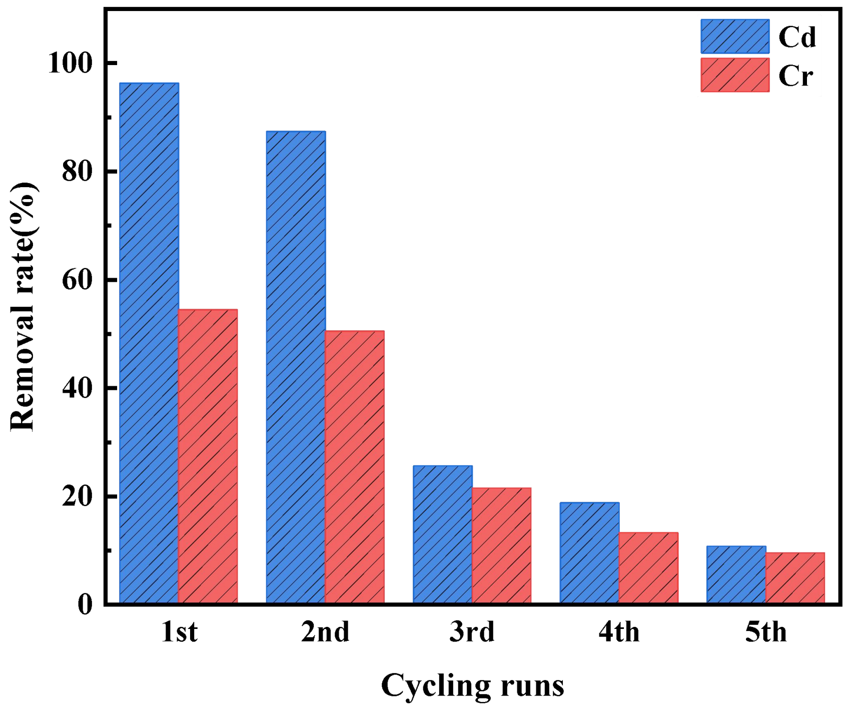

Figure S8. Material recycling and regeneration performance experiments

Table S1 Adsorption kinetic parameters of NZVI@HA for different treatment systems

System	q_e ($mg.g^{-1}$)	k_1 （min^{-1}）	R_1^2	k_2 ($g.(mg.min)^{-1}$)	R_2^2
Cr、Cd Mixed System—Cd	87.1	-0.0137	0.94103	0.01107	0.99989
Cr、Cd Mixed System—Cr	51.9	-0.01562	0.98888	0.01851	0.99999
Cd System	57.6	-0.0176	0.96434	0.017	0.99995
Cr System	55	-0.01494	0.999377	0.0169	0.9987

Table S2 Element contents analyzed by EDS of Cr/CdMC and NZVI@HA+Cr/CdMC systems after reaction

Sample	C/%	O/%	Cr/%	Fe/%	Cd/%
Cr/CdMC	73.39	25.12	1.27	0	0.22
NZVI@HA+Cr/CdMC	58.23	25.92	3.31	6.96	5.58

Table S3 Alpha diversity index of microbial communities in NZVI@HA, Cr/CdMC and NZVI@HA+Cr/CdMC systems

Sample	Chao1	Faith_pd	Observed_species	Pielou_e	Shannon
Cr/CdMC	151.15±11.24[b]	34.01±2.58[b]	107.2±12.08[b]	0.40±0.02[a]	2.72±0.01[b]
NZVI@HA+Cr/CdMC	742.81±10.58[a]	73.74±3.46[a]	517±18.54[a]	0.27±0.01[b]	2.46±0.01[a]
